# Chemokines in the tumor microenvironment: implications for lung cancer and immunotherapy

**DOI:** 10.3389/fimmu.2024.1443366

**Published:** 2024-07-16

**Authors:** Haebeen Jung, Silke Paust

**Affiliations:** The Jackson Laboratory for Genomic Medicine, Farmington, CT, United States

**Keywords:** chemokines, immune cells, cancer, tumor microenvironment, immunotherapy, lung cancer

## Abstract

The tumor microenvironment (TME) is a complex interconnected network of immune cells, fibroblasts, blood vessels, and extracellular matrix surrounding the tumor. Because of its immunosuppressive nature, the TME can pose a challenge for cancer immunotherapies targeting solid tumors. Chemokines have emerged as a crucial element in enhancing the efficacy of cancer immunotherapy, playing a direct role in immune cell signaling within the TME and facilitating immune cell migration towards cancer cells. However, chemokine ligands and their receptors exhibit context-dependent diversity, necessitating evaluation of their tumor-promoting or inhibitory effects based on tumor type and immune cell characteristics. This review explores the role of chemokines in tumor immunity and metastasis in the context of the TME. We also discuss current chemokine-related advances in cancer immunotherapy research, with a particular focus on lung cancer, a common cancer with a low survival rate and limited immunotherapy options.

## 1 Introduction

Chemokines, also known as chemotactic cytokines, are small secreted proteins that play a crucial role in controlling the migration of immune cells to specific tissues (1). Chemokines interact with seven transmembrane G protein-coupled receptors, initiating intracellular signaling that governs cell polarization, adhesion, and movement. Structurally, chemokines are categorized into four families: CXC, CC, CX3C, and C, which are named for the number and location of cysteine residues (C) on the amino terminus of the protein. Cell type-specific epigenetically regulated chemokine receptor expression by distinct leukocyte populations ensures that chemokine gradients can regulate the influx of immune effector cells to sites of inflammation, or, during homeostasis, immune cells to their respective resident tissues (2). Cancer is one of the situations wherein chemokines exert their influence, taking place primarily within the tumor microenvironment (TME).

Cancer immunotherapy has emerged as a significant treatment modality, sparking heightened interest in cancer immunity research. Within the field, there has been an increased interest in the use of immune-related signaling proteins like cytokines and chemokines, as well as diverse immune checkpoint molecules, and the identification of neoantigens (3). These elements have been evaluated as pivotal targets for therapeutic interventions and can also utilized as biomarkers in cancer immunotherapy evaluations. While adoptive cell therapy has demonstrated remarkable success in treating hematologic cancers, for most solid tumors, including lung, pancreatic, breast, and liver cancers, effective cancer immunotherapies have yet to be developed, or if a therapy is available, tumors have proven resistant to treatment (4). This resistance is largely attributed to the intricate and dynamic nature of the tumor microenvironment specific to solid tumors, which presents a significant barrier to effective immune attack (5).

Lung cancer is the second most common cancer and the leading cause of cancer-related deaths worldwide (6). Lung tumors are broadly classified into two categories: non-small cell lung cancer (NSCLC), which account for approximately 80–85% of all lung cancer cases, and small cell lung cancer (SCLC), which represent the remaining 15% of occurrences. NSCLC can be further categorized into adenocarcinoma, squamous cell carcinoma, and large cell carcinoma (3). Unfortunately, the survival rates for metastatic lung cancer, including both NSCLC and SCLC, are generally poor, with a five-year survival rate of only around 4% (7).

Recent technological advancements have enhanced our comprehension of the intricate molecular mechanisms underlying the immunogenicity of lung cancer. Consequently, various immunotherapies, such as therapeutic vaccines, immunomodulators, and monoclonal antibodies targeting checkpoint inhibitors have emerged for lung cancer management. Nonetheless, each approach comes with unique benefits and caveats, prompting the exploration of combined therapies and immunotherapy enhancers (7). Further investigation is needed to identify the optimal combination of immunotherapies, with the potential inclusion of chemokines in this endeavor.

This review will explore the role of chemokines in modulating immune cells within the TME. We will describe how chemokines regulate various immune cell types and facilitate interactions that impact tumor growth and metastasis. Additionally, we will discuss potential strategies for leveraging chemokines in cancer therapy. Finally, we will examine lung cancer-specific chemokine research, including basic and pre-clinical studies, as well as ongoing clinical applications that focus on TME immunity.

## 2 Chemokines in the TME

**Figure 1:**
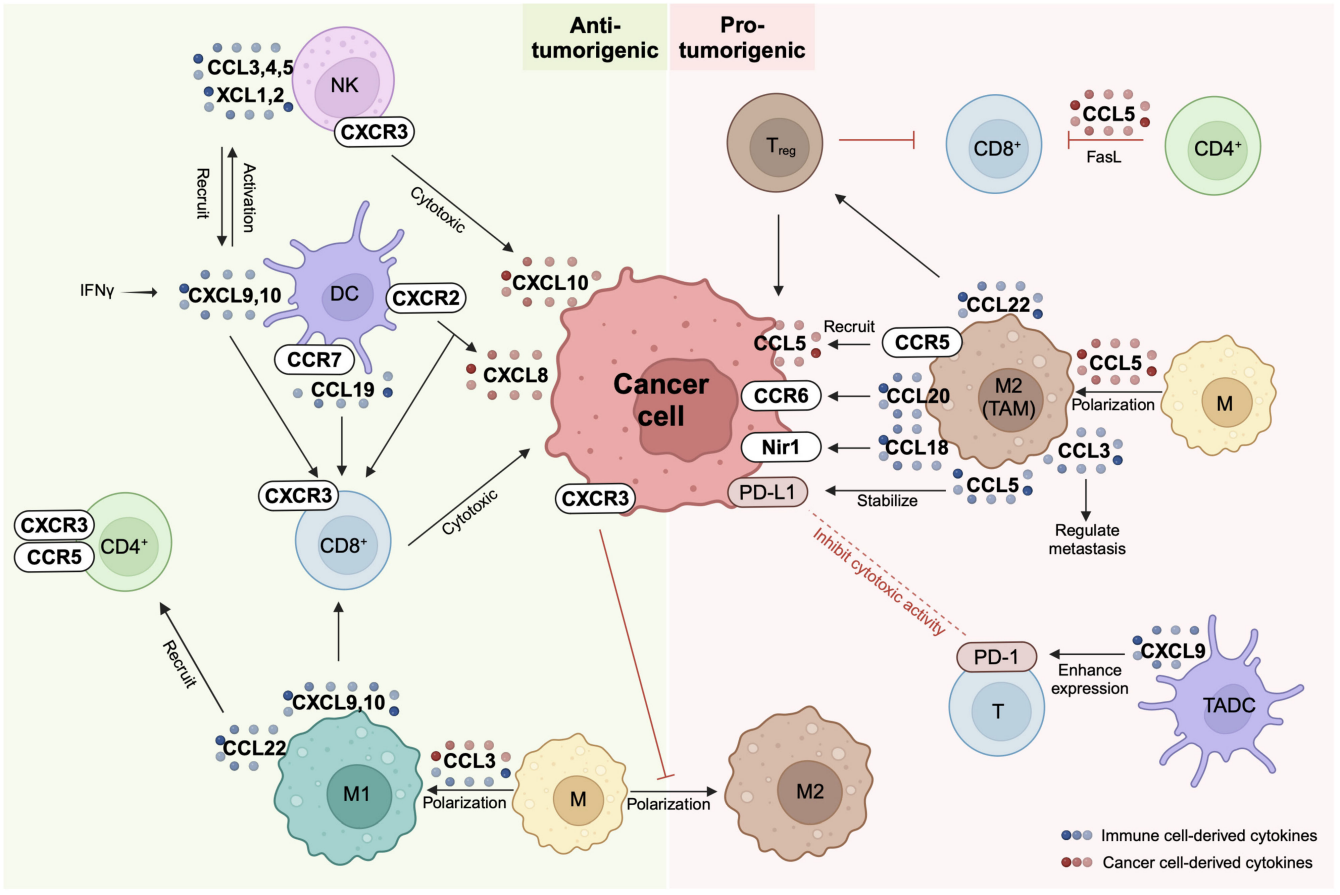
Chemokine network between immune cells and cancer cells. Chemokine receptors (CCR or CXCR) and their ligands (CCL or CXCL) are expressed on cancer cells and a variety of immune cells including natural killer (NK) cells, dendritic cells (DC), macrophages (M), and T cells. The same chemokine can exhibit either anti-tumorigenic or pro-tumorigenic properties depending on the specific cell type it interacts with. For instance, macrophages display anti-tumorigenic characteristics when polarized to M1 and pro-tumorigenic attributes when polarized to M2. NK cells and CD8+ T cells contribute to cancer cell elimination through cytotoxic actions, representing pivotal players in the immune response against tumors. On the other hand, PD-1/PD-L1 interactions occurring between cancer cells and T cells serve to suppress T cell activity, contributing to immune evasion. Additionally, regulatory T (Treg) cells, tumor-associated dendritic cells (TADC), and M2 macrophages collectively contribute to the establishment of an immunosuppressive environment within the tumor microenvironment. These intricacies highlight the complex interplay between various immune cell types and the tumor cells in shaping the dynamics of cancer progression and response to immunotherapy. Created with BioRender.com.

The TME is a complex interconnected network of immune cells, fibroblasts, blood vessels, and extracellular matrix surrounding the tumor and involved in tumor growth and metastasis (8, 9). The genetic, immunologic, and metabolomic diversity of the TME can result in varied treatment outcomes within cancer and be a major cause of therapy resistance (10, 11). Chemokines are involved in regulating immune cell infiltration and shaping tumor progression. Since chemokines are pleiotropic, their combinatorial intratumoral expression can have complex and diverse tumor-promoting or tumor-fighting effects (**Figure 1**). Therefore, depending on the type of tumor and its immune infiltration, it is necessary to individually assess whether chemokine signaling promotes or inhibits tumor growth.

### 2.1 Impact of chemokines on tumor immunity

**Figure 2:**
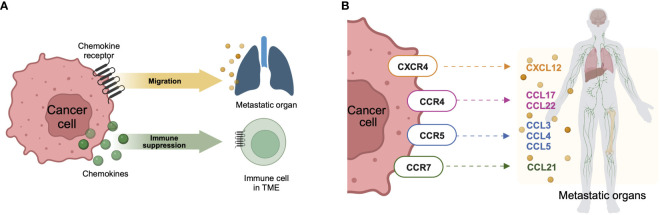
Chemokine network in metastasis. **(A)** When cancer cells express chemokine receptors, their role extends to facilitating migration towards chemokine ligands expressed in metastatic organs. In this context, these receptors play a crucial role in directing the movement of cancer cells within the body. Conversely, when cancer cells express chemokine ligands, their impact is primarily on the immune cells within the tumor microenvironment, contributing to the establishment of immunosuppression. This dual interaction underscores the intricate balance between cancer cells and the surrounding microenvironment, influencing both the metastatic potential of the cancer and the immune response within the tumor. **(B)** Chemokine receptor-ligand axes are involved in metastasis. These specific axes of chemokine receptors expressed on cancer cells and chemokine ligands expressed in metastatic organs are commonly involved in the migration of cancer cells, leading to metastasis. Created with BioRender.com.

Immune cells such as natural killer (NK) cells, dendritic cells (DCs), macrophages, and T cells are crucial tumor-fighting components of the TME (12). However, cancer cells also secrete chemokines, altering the TME and its immune cell composition to be more tumor-promoting (13) (**Figure 2A**). The underlying mechanisms by which these immune cell populations and cancer cell-secreted chemokines interact are complex, and understanding them is crucial for cancer research progress (14, 15). In this section, we will discuss how individual chemokines modulate the innate and adaptive immune response in healthy and malignant tissues, before discussing strategies to use chemokines in cancer therapy in section 3, and effects of chemokines on lung cancer immunity in section 4.

#### 2.1.1 NK cells

NK cells are a type of cytotoxic lymphocyte of the innate immune system. NK cells are inhibited by the robust expression of major histocompatibility complex class (MHC) I molecules, which mark potential target cells as ‘self’ and trigger inhibitory killer cell immunoglobulin-like receptor signaling to prevent NK cell activation. Cells with low MHC-I expression are considered as ‘missing self’ and are preferred targets for NK cell-mediated cytotoxicity and other effector functions (16), provided NK activating signals (*e.g.*, in the form of stress ligands) are also present. However, malignant cells often upregulate stress ligands and down-regulate MHC-I to escape cytotoxic T-cell immunity, thereby becoming ‘missing self’ targets. Thereby, NK cells play an important role in early cancer defense. Chemokine signaling can augment anti-tumor immunity, as C-X-C motif chemokine receptor (CXCR) 3 expressed on NK cells senses C-X-C motif chemokine ligand (CXCL) 10 secreted by cancer cells and exerts cytotoxic activity at the cancer site (17). In addition to their direct anti-tumor functions, NK cells secrete chemokines to recruit key immune cell populations required for robust anti-tumor immunity. Indeed, intra-tumoral cytotoxic NK cells express high levels of C-C motif chemokine ligand (CCL) 3, CCL4, CCL4L2, and CCL5 transcripts, while additional NK cell subsets express X-C motif chemokine ligand (XCL) 1 and XCL2 transcripts (18). When secreted by intratumoral NK cells, XCL1, XCL2, and CCL5 serve as chemoattractants for type 1 conventional DCs (cDC1s), augmenting the recruitment of cDC1s into the TME where these powerful antigen-presenting cells can activate the cytotoxic CD8^+^ T lymphocytes (CTLs) for tumor attack (19, 20). In addition to aiding cDC-CTL interactions, Interleukin (IL)-18-primed “helper” NK cells secrete CCL3 and CCL4 to attract immature DCs to stimulate intratumoral CD8^+^ T cells via CCL5, CXCL9, and CXCL10 (21).

#### 2.1.2 DCs

DCs are professional antigen-presenting cells (APCs) bridging innate and adaptive immunity by initiating immune tolerance and antigen-specific immunity (22). In tumors, DCs present tumor antigens to elicit an antigen-specific T-cell response (23). The CXCL9/CXCL10-CXCR3 axis is crucial for NK cell-DC-CD8^+^ T cell crosstalk and anti-tumor immune response (24). CD103^+^ DCs recruit NK cells by releasing CXCL9, inducing the upregulation of the NK cells-activating receptor NKG2D while downregulating the inhibitory NK cell-expressed receptor NKG2A (25). The expression of CXCL9 in DCs is induced by interferon-gamma (IFN-γ) (26) and can be further upregulated with immune checkpoint therapies targeting TIM-3 (27) or CD47 (25). However, when secreted by tumor associated-dendritic cells (TADCs), CXCL9 can also enhance the expression of the checkpoint molecule ligand programmed death-ligand 1 (PD-L1), thereby attenuating anti-tumor T cell immunity via the PD-1/PD-L1 pathway (28). The CCL19/C-C motif chemokine receptor (CCR) 7 axis poses an anti-cancer property that both CCL19^+^ DCs (29) and CCR7^+^ DCs (30) augment CD8^+^ T cell immunity. Lastly, CXCR2 expression by DCs induces their migration into the tumor through the CXCL8-CXCR2 axis (31, 32), since CXCL8, a CXCR2 ligand, is expressed by endothelial cells, tumor-associated macrophages (TAMs), and cancer cells. This CXCL8-CXCR2-mediated recruitment of DCs towards the tumor site induces DC activation and CD8^+^ T cell infiltration.

#### 2.1.3 T cells

T cells are an essential component of adaptive immunity. Once primed and activated by APCs, T cells migrate to the tumor site and exert antitumor activity in a process called chemotaxis, which, as its name implies, is triggered by chemokines (33). CCL5 and the IFNγ-inducible chemokines CXCL9/10/11 are critical components of this process. Tumor-derived CCL5 and APC-derived CXCL9 enhance CD8^+^ T cell infiltration in solid tumors; this effect is more notable for CXCL9 (26, 34, 35). CD4^+^ Th1-polarized effector memory cells expressing CXCR3 and CCR5, the receptors for CXCL9/10/11 and CCL5, respectively, are another significant component of tumor-infiltrating lymphocytes (TILs). Indeed, the proportion of intratumoral CCR5^+^CXCR3^+^CD4^+^ cells has been observed to be inversely proportional to metastasis formation in renal carcinoma patients (36). In addition, CD40-signaling-induced CCL5 elicits tumor infiltration by CD4^+^ T cells and enables immunosuppression of cancer growth (37). In pancreatic ductal adenocarcinoma, CCL4, CCL5, CXCL9, and CXCL10 are directly associated with CD8^+^ T-cell infiltration (38). However, CCL5 can also suppress T cell immunity at the tumor site and may even promote tumor immune evasion (39) by inducing tumor infiltration by regulatory T (Treg) cells, impairing the cytotoxic activity of CD8^+^ T cells (40). In addition, cancer cells can stimulate CD4^+^ T cells to secrete CCL5, inducing Fas-mediated apoptosis of CD8^+^ T cells (41, 42).

#### 2.1.4 Macrophages

Alongside DCs, macrophages are major APCs and are an important component of innate immunity. Macrophages are plastic cells that can be polarized into two phenotypes: pro-inflammatory M1 macrophages and anti-inflammatory/immunosuppressive M2 macrophages. Tumor-associated macrophages (TAMs) are similar to M2 macrophages, except that TAMs express Fc receptors for IgG, C-type lectin, and heat shock proteins and secrete CCL2 and CCL5 (43). TAMs-secreted CCL5 stabilizes tumor-expressed PD-L1, inhibiting the cytotoxic activity of T cells and inducing immune escape (44). On the other hand, cancer cell-secreted CCL5 induces the polarization of monocytes into M2 macrophages and recruits CCR5^+^ TAMs (45–47). Another chemokine axis regulating TAM activity is CCR6-stimulation by CCL20. Circular RNAs secreted by cancer cells stimulate TAMs to secrete CCL20 (48), increasing TAM migration and the invasion of CCR6-expressing tumors while also inducing epithelial-mesenchymal transition (EMT) (49, 50). The CXCL9/CXCL10-CXCR3 is another axis with anti-tumor properties. Following dual PD-1/CTLA-4 blockade, macrophages secrete CXCL9 and CXCL10, increasing tumor infiltration of CD8^+^ T cells (51). Furthermore, the expression of CXCR3 on cancer cells reduces polarization into TAMs; a study of gastric cancer patients demonstrated that those who expressed more CXCR3 had a better prognosis (52). Lastly, previous studies of CCL3 (a CCR5 ligand) and CCL22 (a CCR4 ligand) have shown conflicting results. While TAMs induce cancer cell migration and regulate metastasis by secreting CCL3 (53), docetaxel, a classical anti-mitotic chemotherapy drug, triggers the secretion of CCL3 from macrophages and cancer cells and induces polarization towards M1 macrophages, subsequently facilitating cancer cell phagocytosis (54). Similarly, macrophage-derived CCL22 can either suppress tumor immunity by increasing Treg cell tumor infiltration (55) or promote tumor immunity by recruiting helper T cells (56).

### 2.2 Metastasis regulation by chemokines and chemokine receptors

Some chemokines and their receptors aid in TME formation by promoting tumor cell proliferation and metastasis. Since metastatic cancer has a low chance of remission, and most cancer-related deaths result from metastatic cancer rather than primary cancer (57), chemokines responsible for metastasis can be potential therapeutic targets or prognostic markers (58, 59). Although most chemokine receptors can be expressed on both immune and cancer cells, in this review, we focus on cancer cell-expressed chemokine receptors because of their ability to increase a tumor’s metastatic potential by inducing EMT when chemokine receptor-expressing cancer cells migrate to metastatic sites rich in chemokine ligands (**Figure 2B**).

CXCR4 is the receptor for CXCL12, also called stromal cell-derived factor 1 (SDF1), and is the most widely expressed chemokine receptor in human cancer (60). CXCR4 is highly expressed by human cancer cells and is involved in metastasis formation, as confirmed experimentally by the *in vivo*-neutralization of CXCR4 (61). Studies in solid tumors, as such the depletion of the transcription factor forkhead box P3 in mammary epithelial cells (62), the overexpression of transcription factor 12 in hepatocellular carcinoma (63), and the acetylation of transcription factor Krüppel-like factor 5 in prostate cancer (64) have been performed to evaluate the effects of CXCR4 modulation on tumor growth. Additionally, microRNA-sequencing from SCLC patient serum revealed that miR-1 expression reduces tumor growth and metastasis by targeting the CXCR4/FOXM1/RRM2 axis (65). Upregulation of CXCR4 increases chemotaxis of tumor cells towards pro-metastatic CXCL12 promoting metastasis in solid tumors, as well as tumor angiogenesis and tumor growth through the activation of the mitogen-activated protein kinase/extracellular signal-regulated kinase (MAPK/ERK) and phosphatidylinositol 3-kinase/Ak strain transforming (PI3K/AKT) signaling pathways (63, 66–68). The CXCL12-CXCR4 axis is also related to cisplatin-induced metastasis which is known to be the long-term detrimental effect of platinum-based chemotherapy (69). Cisplatin activates the CXCL12-CXCR4 axis and induces lung metastasis by regulating the expansion of inflammatory monocytes in mouse model of lung cancer metastasis (69). Platinum-treated clinical samples of NSCLC show elevated CXCL12 levels, which were associated with worse clinical outcomes (69).High levels of CXCL12, the ligand for CXCR4, have been found in metastasis in organs such as the lung, liver, bone marrow, and lymph nodes through the recruitment of CXCR4-expressing cancer cells (63, 70–72).

CCR4 and its ligands CCL17 and CCL22 are also involved in cancer cell migration. The expression of CCL17 and CCL22 is increased in the lung, liver, and brain of tumor-bearing nude mice with an increased malignancy phenotype of cancer cells (73). CCR4 was highly expressed by melanoma cells in brain metastatic regions with altered AKT phosphorylation patterns (74, 75). Moreover, CCR4 facilitated metastasis through the ERK signaling pathway in colorectal cancer (76), bladder cancer (77), hepatocellular carcinoma (78), and lung cancer (79). As such, CCR4 targeting could enable novel cancer immunotherapies for solid tumors.

The chemokine receptor CCR5 binds with affinity to CCL3, CCL4, CCL5, and CCL8. Among CCR5 ligands, its interaction with CCL5 has been studied the most. These studies established the CCR5-CCL5 axis’s ability to drive cancer progression and recruit tumor-infiltrating leukocytes in several cancer types (80). CCR5 is overexpressed in lymphoma (81, 82), hepatocellular carcinoma (83), pancreatic cancer (80), colorectal cancer (84), and many other cancers (85–87). In addition, high tissue or plasma levels of CCL5 correlate with unfavorable outcomes in pancreatic cancer patients (80). Through PI3K/AKT signaling pathways, the CCL5-CCR5 axis induces cancer cell survival, invasion, migration, and metastasis (83, 85, 87). As part of the CCR5 metastatic process, the CCL3-CCR5 (88, 89) and the CCL4-CCR5 (90, 91) axes also drive cancer cell invasion and migration.

Another receptor involved in metastasis is CCR7. CCR7 is crucial for immune cell homing of immune cells to lymphoid organs and is normally expressed on mature DCs and naïve lymphocytes (92). Due to their lymphoid origins, many leukemias and lymphomas also express CCR7 (93), enabling lymph node metastasis (94, 95). Of the two ligands of CCR7, CCL19, and CCL21, only CCL21 is associated with metastasis, contributing to lymphatic metastasis in pancreatic (94), lung (95–97), breast (61, 98), and other cancers (99–102) via ERK signaling. Moreover, CCR7 enhances angiogenesis by increasing vascular endothelial growth factor (96, 102, 103). Tumor necrosis factor -α, a proinflammatory cytokine, can increase CCR7 expression in cancer cells (104), promoting the production of CCL21 in human lymphatic endothelial cells (97).

Other chemokine axes, such as the CXCL8-CXCR1/2 axis (105, 106), the CXCL9-CXCR3 axis (107, 108), the CXCL13-CXCR5 axis (109), the CCL2-CCR2 axis (110, 111), and the CCL20-CCR6 axis (112, 113), are also involved in cancer metastasis; the chemokine axis can vary depending on the type of cancer (114–116). The wide variety of chemokine receptors involved in metastasis underscores the importance of assessing changes in chemokine expression as part of cancer treatments to predict cancer and metastasis progression and to inform treatment options.

## 3 Strategies to use chemokines in cancer therapy

Chemokines have been validated as disease targets through genetic depletion and antibody neutralization. Chemokine receptors are also among the most structurally well-studied class A family of G protein-coupled receptors, leading to their use in many drug discovery programs (117). However, using a chemokine itself as a drug presents particular challenges. One major difficulty lies in the complexity and high redundancy of the chemokine system. Chemokines can bind to multiple receptors and act as an agonist for one receptor while acting as an antagonist for another. Similarly, a chemokine receptor can have an affinity for various chemokines exhibiting biased signaling. Moreover, a cell can express several chemokine receptors, varying their expression by disease state (118). In addition, chemokine receptors can form homo- and hetero-dimers, making it difficult to develop drugs that precisely target isolated chemokine/chemokine receptor pathways (119). These considerations make the pharmaceutical targeting of chemokine receptors challenging (117). Indeed, adverse effects from the use of chemokine antagonists have been reported: the clinical trial of aplaviroc, a CCR5 antagonist, was terminated prematurely due to compound-induced liver toxicity (120).

Nevertheless, using chemokines in cancer therapy can be valuable in guiding immune cells and cancer cells to specific locations, influencing whether the TME promotes or suppresses tumor growth. Efforts are ongoing to address the challenges associated with the development of chemokine drugs and therapies. These may include combining chemokines with other existing therapies, modification of chemokine expression by immune cells, or utilizing chemokines for tumor targeting.

### 3.1 Adoptive cell therapies

Adoptive cell therapies, such as chimeric antigen receptor (CAR)-T cell therapy and NK cell-based immunotherapy, have proven to be revolutionary treatments for certain subsets of B cell leukemia and lymphoma (121). Over the past decade, adoptive cell therapies have been investigated for the treatment of solid tumors. However, the anti-cancer activity of infused immune cells can be limited at least in part by a lack of penetrance into the solid tumor and the tumor’s immunosuppressive environment (4), and in the case of CAR-T cell-therapy, limited tumor neoantigen availability, and insufficient CAR-T cell infiltration of the tumor and metastatic tissues (5). To overcome these limitations, strategic expression of chemokine receptors can be used to augment solid tumor immunotherapy. An example of this is the forced expression of the CCL2 receptor CCR2b in B7-H3-specific CAR-T cells, which significantly enhanced the anti-tumor activity to brain metastases in mouse xenograft models by enabling CAR-T cells to effectively cross the blood-brain barrier (122). Similarly, the expression of IL-7 and CCL19 improved the anti-tumor potential of CAR-T cells by augmenting their activation and boosting the generation of memory responses for both recipient conventional T cells and administered CAR-T cells against mastocytoma in mice (123).

NK cells are also being developed as immunotherapy infusion products (124) as an alternative to CAR-T cell therapies with significant toxicity issues (121). CCR7-expressing CAR NK cells were shown to be more effective than control CAR-NK cells in controlling tumors via the CCL19-CCR7 axis both *in vitro* and in a murine lymphoma xenograft model (125). Further, CXCR4 expressing CAR-NK cells exhibited an enhanced ability to migrate towards a CCL12 gradient while maintaining functional cytolytic activity towards target cancer cells (CD19^+^ Nalm-6 cells) *in vitro* (126). In addition to directly expressing chemokines in CAR-engineered cells, chemokines can also be used to indirectly enhance the effectiveness of CAR cell therapy. For example, CXCL11-armed oncolytic adenoviruses were used as an adjuvant in CAR-T cell therapy, reprogramming the immunosuppressive TME in a syngeneic glioblastoma mice model (127).

Another approach is to regulate the endogenous gene expression in immune cells to modulate the expression of chemokines, followed by adoptive cell therapy. To exploit the CCL5-CCR5 axis that allows NK cell infiltration of tumors, researchers have used oncolytic vaccinia virus Western Reserve strain to increase CCR5 expression on NK cells and CCL5 expression on cancer cells (128). Comparisons of NK cells from tissues of patients with various cancers and healthy individuals showed that NK cells from cancer patients had lower expression of CCR5. When NK cells were genetically engineered to overexpress CCR5 and then infused, survival rates increased in mice. Simultaneously increasing CCL5 expression on cancer cells increased the anti-cancer effect even more (128). In a different study, NK cells were engineered to overexpress CCR2B and CCR4 to induce migration toward CCL2- and CCL22-expressing tumor cells, respectively (129). Also noteworthy is that in both studies, NK cells were genetically engineered without their functional impairment.

### 3.2 Combination of chemokines with immune checkpoint inhibitors (ICIs)

ICIs are powerful treatments that have expanded the field of cancer immunotherapy to allow the treatment of solid malignancies. Currently, FDA-approved ICIs include pembrolizumab, nivolumab, cemiplimab, dostarlimab, toripalimab, retifanlimab (PD-1-specific antibodies), ipilimumab, tremelimumab (CTLA-4-specific antibody), atezolizumab, avelumab, and durvalumab (PD-L1-specific antibodies) (130). However, treatment efficacy still varies between patients. For example, in solid tumors, such as NSCLC, ICIs can be used when other therapies have failed, with conflicting long-term results. While some studies have shown no benefit to long-term ICI treatments (131, 132), others have reported positive outcomes with pembrolizumab two (133) and five years post-treatment (134). Because the TME can play a role in modulating ICI efficacy, chemokines can be used to overcome TME-induced immunosuppression and increase the response rate to ICI (135). One example of this is a study of 27 NSCLC patients, in which a positive correlation was identified between the patients’ CXCL13 expression and observed ICI efficacy. In a subsequent animal model-based mechanistic study administering recombinant CXCL13 to mice with lung cancer, it was demonstrated that CXCL13 treatment enhanced therapeutic PD-1 blockade by increasing antigen-experienced T cell subsets (135). Another example of this is a phase IIa clinical trial (NCT02826486) of a combination of the CXCR4 antagonist BL-8040 and pembrolizumab in patients with metastatic pancreatic duct adenocarcinoma (PDAC), where BL-8040 increased CD8^+^ effector T cell tumor infiltration, decreased myeloid-derived suppressor cells (MDSCs), and further decreased circulating Treg cells, resulting in a favorable outcome (136).

### 3.3 Use of nanoparticles for chemokine delivery

Researchers have developed nanoparticles to enhance the delivery of chemokines in cancer therapy while performing multiple functions. For example, using NPs to deliver maraviroc, a small molecule inhibitor of CCR5, improved bone marrow residence time while reducing leukemic burden compared to treatment with maraviroc alone in mouse models (137). NPs have been developed using different approaches to enable the use of CXCR4 in cancer therapy. For example, the AMD-NP-PTX nanocomplex was developed for targeting ovarian cancer (136). It contains a small molecule antagonist for CXCR4 (AMD3100) and paclitaxel (PTX), a common chemotherapy drug. Due to the overexpression of CXCR4 in ovarian cancer, AMD-NP-PTX can be effectively delivered to the cancer site, inhibiting the CXCL12-CXCR4 axis and exerting chemotherapeutic activity with reduced off-target toxicity. Similarly, AMD31100 coated on synthetic protein nanoparticles (AMD3100-SPNPs) delivered to a glioblastoma mouse model inhibited tumor proliferation and reduced infiltration of CXCR4^+^ MDSCs while overcoming poor pharmacokinetic properties of AMD3100 and restoring the blood-brain barrier (138). CXCR4 antagonistic nanoparticles have also been designed to enhance the response rate of anti–PD-L1 therapy to treat lung cancer (139). These nanoparticles increased the effector T cell infiltration in solid tumors by reducing the tumor’s fibrosis and tumor-resident MDSCs and Treg cells, subsequently enhancing the effectiveness of the PD-1/PD-L1 immunotherapy both *in vivo* and *in vitro*. Alternatively, NPs can be used to upregulate chemokine-related genes in cells rather than to directly deliver chemokine-related drugs to the tumor. Polymeric NPs containing CXCR4 DNA were used to upregulate CXCR4 in human adipose-derived stem cells to target tumor hypoxia (140). In another study, a hydrogel nanoparticle complex, LPR@CHG, containing lipid-immune regulatory factor 5 mRNA/CCL5 siRNA (LPR) nanoparticle complexes coated with chitosan/HTCC/glycerophosphate was designed as a potential pancreatic cancer treatment (141). This hydrogel complex downregulated CCL5 secretion of tumor cells, which contributed to an increase in M1 macrophages and elicited a T cell-mediated immune response, ultimately controlling pancreatic cancer in mice.

## 4 The effects of chemokines on lung cancer immunity

**Figure 3:**
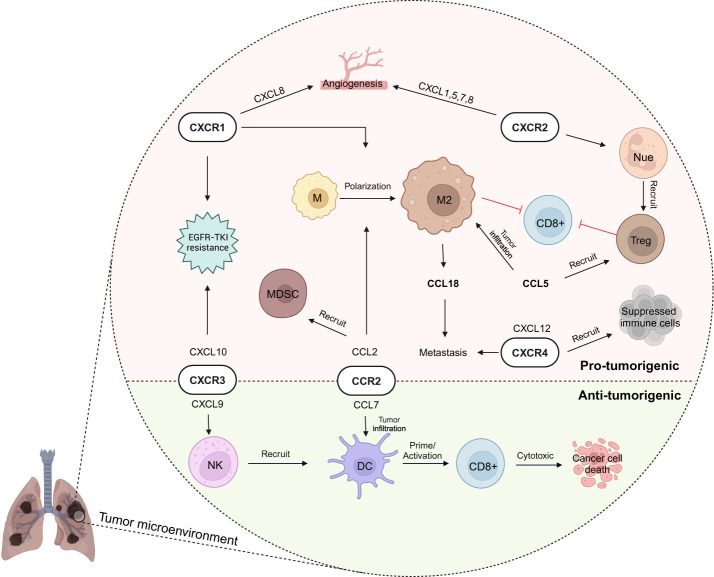
Chemokine network in lung cancer immunity. In lung cancer’s tumor microenvironment (TME), chemokines play a dual role, fostering both anti-tumor immunity and pro-tumorigenic activities (e.g., CXCR3 and CCR2 involvement with cytotoxic activity by natural killer (NK) cell - dendritic cell (DC) -T cells as well as immunosuppressive activity). Immunoregulatory cells like M2 macrophage, regulatory T (Treg) cell, and myeloid-derived suppressor cell (MDSC) are recruited by chemokines to the TME, creating an immunosuppressive environment. Meanwhile, CXCR1 and CXCR2 contribute to pro-tumorigenic angiogenesis, supporting cancer growth and migration to other organs. These complexities highlight the need for targeted approaches to modulate immune responses effectively in lung cancer. EGFR-TKI, epidermal growth factor receptor tyrosine kinase inhibitor; M, macrophage; Nue, neutrophil. Created with BioRender.com.

Lung tissue represents a unique microenvironment supportive of primary lung carcinoma development and metastases originating from tumors outside the lung (142). Recent advances in understanding the tumor-reprogrammed microenvironment have led to the development of targeted therapies for lung cancer. Targeting angiogenesis and immune cells has shown promise, sparking interest in understanding other TME components to improve clinical outcomes in lung cancer (142). In this context, chemokines affect tumor growth, metastasis, and even the effectiveness of radiation therapy by involving various cellular interactions and altering TME (143). In this section, we will discuss chemokine networks in lung TME (**Figure 3**), highlighting recent chemokine-related advances in lung cancer immunotherapy.

### 4.1 Preclinical studies of chemokines in lung cancer

In a study of NSCLC patients with metastatic disease, a high expression of CXCR1 was associated with a poor prognosis reflected by the patient’s tumor node metastasis (TNM) stage (144). The expression of CXCR1 was positively correlated with tumoral neutrophils and macrophages and with the polarization of macrophages to the immunosuppressive M2 phenotype. Furthermore, patients resistant to epidermal growth factor receptor tyrosine kinase inhibitor (EGFR-TKI) treatment expressed high levels of CXCR1. In these patients, CXCR1 modulated the tumor microenvironment as well as the PI3K/AKT and ERK pathways, which are shared by CXCR1 and EGFR-TKI. These findings suggest that CXCR1 could be a therapeutic target for NSCLC.

CXCR2 is mainly expressed by neutrophils and associated with pro-tumorigenic properties, especially angiogenesis. Among the ligands of CXCR2, CXCL1, 5, 7, and 8 are considered major angiogenic chemokines in NSCLC (145), with the overall level of angiogenic chemokines being the strongest predictor of tumor vessel density in NSCLC (146). Additionally, lung tumors in CXCR2^−/−^ mice presented increased necrosis and reduced vascularity (147). Indeed, tumor-infiltrating CXCR2^+^ neutrophils play a significant role in shielding tumor cells from CD8^+^ T and NK cell-mediated cytotoxicity. Their presence contributes to the recruitment of Treg cells and facilitates tumor cell metastasis. In a murine lung cancer model, CXCR2^+^ neutrophils TGF-β and Arg-1 were significantly increased, causing immunosuppression and allowing tumor cells to escape immune attack. In contrast, CXCR2 inhibition reduced neutrophil infiltration and promoted CD8^+^ T cell activation (148). Single-cell RNA analysis of human lung squamous cell carcinoma (LUSC) tissues also showed an increase in these tumor-infiltrating neutrophils, characterized by CXCL8-CXCR2 expression (149). In addition, CXCR2 regulated Treg cell migration into malignant pleural effusions (MPEs) via the miR141-CXCL1-CXCR2 pathway, decreasing the median survival of NSCLC patients with MPE (150).

Despite the important role of the CXCL9/CXCL10-CXCR3 axis in NK/DC/CD8^+^ T cell crosstalk, this pathway has been insufficiently studied in the context of lung cancer. What we do know is that the commensal microbiota may influence the development of lung cancer in a *Kras*-driven mouse model, where an antibiotic-treated group exhibited high expression of CXCL9 and CXCR3, resulting in increased recruitment of NK cells and CD8^+^ T cells to the tumor, highlighting the importance of the CXCL9-CXCR3 axis in lung cancer immunity (151). However, a different study revealed that CXCL10-CXCR3 induced resistance to EGFR-TKI treatment in a transgenic lung cancer mouse model (152). During early EGFR-TKI treatment, increased CXCL10 levels stimulated oncogenic signaling in persisting tumor cells, contributing to EGFR-TKI resistance through autocrine and paracrine pathways. These contrasting results highlight the need for additional work to address the contributions of the CXCL9/CXCL10-CXCR3 to lung cancer immunity.

High CXCR4 expression in NSCLC including lung adenocarcinoma (LUAD) and LUSC is correlated to poor patient prognosis (153, 154). Paradoxically, the CXCR4^high^ NSCLC tissues recruit more immune cells into NSCLC tissues with increased immune checkpoint expression and bring a higher response rate to immunotherapy compared to the CXCR4^low^ NSCLC tissues (153). Furthermore, the CXCR4 antagonists suppressed the growth of lung cancer growth: Peptide R prevented the recruitment of metastasis-initiating cells and inflammatory monocytes toward CXCL12-enriched sites (69) and AMD3100 reduced the progression of cancer in orthotopic SCLC mouse model (155).

Another critical chemokine axis is the CCL2-CCR2 pathway, which contributes to the polarization of macrophages toward an M2 phenotype. Tumor macrophage infiltration and CCR2 expression have been found to correlate with both tumor stage and metastasis in human lung cancer samples (156). Decreased expression of CCL2 in human SCLC (157) and blockage of CCL2 in a mouse lung cancer model (158) showed lower macrophage infiltration of tumors with a reduction in M2 polarization and, as a consequence, an increase in CD8^+^ T cell activation. Therapeutic CCL2-blockade also decreased the recruitment of MDSCs in both the blood and tumors collected from lung cancer-bearing mice (159) and resulted in increased CD4^+^ and CD8^+^ T cell infiltration of tumors, higher production of IFNγ, and improved survival of tumor-bearing mice. These effects were even more pronounced when combined with PD-1-specific ICI therapy.

CCL7, another ligand of CCR2, has been studied in depth in solid tumors. Although high CCL7 expression in colorectal (160, 161), breast (162), and uterine (163) cancer is known to promote metastasis and suppress tumor immunity, the expression of CCL7 in NSCLC has been shown to be anti-tumorigenic (164). CCL7 is highly expressed by NSCLC tumors and positively correlated with cDC1 infiltration and overall survival in NSCLC patients. In LUAD mouse models, alveolar macrophage-derived CCL7 increased infiltration of cDC1s into the TME and potentiated the proliferation of intra-tumoral T cells. Similarly, CCL7 administration prolonged the survival of mice with lung cancer and enhanced the efficacy of PD-1 ICI. This study is consistent with the idea that chemokines may have different roles in different types of cancer and suggests that CCL7 has the potential to be an adjuvant in immune checkpoint therapy for lung cancer (165).

Secretion of CCL5, a ligand for CCR3, CCR5, and CCR1 (80), was markedly increased in mouse lung cancers harboring oncogenic EGFR and KRAS mutations (166). CCL5 deficiency in KRAS mutant LUAD resulted in a decrease in Treg cells and reduced lung tumor burden, indicating that the production of CCL5 by tumor cells contributes to an immunosuppressive environment in the lung. Additionally, high expression of CCL5 was associated with a negative prognosis, Treg cell recruitment, and altered CD8 effector function in LUAD patients. Consistent with this study, the spatial transcriptomic profiles of NSCLC showed upregulation of CCL5 in tumors with high infiltration of CD163^+^ TAMs (167).

Another chemokine associated with poor survival in NSCLC is CCL18, a ligand for the PITPNM3, GPR30, and CCR8 receptors (168). In lung cancer, TAM-derived CCL18 is thought to promote metastasis by facilitating the migration of lung cancer cells (169). Compared to healthy controls, NSCLC patients had higher serum CCL18 concentrations, which were also associated with poor survival (170). Further, local CCL18 concentrations were higher in lymph node-positive NSCLC patients (171), suggesting that CCL18 could potentially be used as an independent diagnostic marker in metastatic NSCLC (172). In addition to CCL18, CXCL12 and CCL22 are known to alter the TME in lung cancer by modulating TAM activity (56, 173).

Overall, CXCR2, CXCR4, CCR2 are currently the most extensively studied chemokine receptors in NSCLC and are considered potential biomarkers for lung cancer (**Table 1**). These chemokine receptors show promise as therapeutics, especially when combined with ICI, as demonstrated in several studies (147–150, 164, 165). The lung is not only a primary tumor site but also a common site for metastasis. Thus, the therapeutic use of chemokines to prevent lung tumors and metastasis would be a positive direction for future cancer research and could potentially offer treatment options for patients who have failed more traditional therapies.

**Table 1:** Pre-clinical studies about chemokine in lung cancer immunity.

Chemokine		Molecule	Cancer Type	Property	Immune cells involved	Reference
Receptor	Ligand					
CXCR1		EGFR-TKI	NSCLC	Pro-tumorigenic	Neu, M2	(144)
CXCR2			NSCLC	Pro-tumorigenic	Neu, CD8^+^ T, NK	(147)
	CXCL8		Lung cancer	Anti-tumorigenic	Neu, CD8+T	(148)
			LUSC	Pro-tumorigenic	Neu	(149)
	CXCL1		NSCLC	Pro-tumorigenic	Treg	(150)
CXCR3	CXCL10		Lung cancer	Anti-tumorigenic	CD8+T, NK	(151)
	CXCL10	EGFR-TKI	Lung cancer	Pro-tumorigenic		(152)
CXCR4			NSCLC	Pro-tumorigenic	Increased immune checkpoint expression	(153)
	CXCL12	Peptide R	Lung cancer	Pro-tumorigenic	Monocyte	(69)
	CXCL12	AMD3100	SCLC	Pro-tumorigenic		(155)
	CXCL12		Lung cancer	Pro-tumorigenic	TAM	(173)
CCR2	CCL2		Lung cancer	Pro-tumorigenic	M2, CD8+T	(156–158)
		ICI	Lung cancer	Pro-tumorigenic	MDSC, CD4+T, CD8+T	(159)
	CCL7	ICI	NSCLC	Anti-tumorigenic	DC, Alveolar M, T	(164)
		ICI	NSCLC	Anti-tumorigenic	DC, T	(165)
	CCL5		LUAD	Pro-tumorigenic	Treg, CD8+T	(166)
			NSCLC	Pro-tumorigenic	TAM	(167)
	CCL18		NSCLC	Pro-tumorigenic	TAM	(169)
	CCL22		Lung cancer	Pro-tumorigenic	TAM	(56)

### 4.2 Clinical trials using chemokine-related therapies in lung cancer

Due to the intricate and diverse characteristics of chemokines, the selection of the appropriate target and therapeutic approach is paramount. Currently, most studies on chemokine therapy remain in the preclinical stage, yet some clinical trials have been undertaken utilizing various chemokine systems and approaches (**Table 2**). For instance, combinations of chemokines for effective immunotherapy are being studied in several clinical trials. There is one Phase I study that engineered anti-glypican-3-CAR-T cells to secrete IL-7 and CCL19 (anti-GPC3–7 × 19 CAR-T) for enhancing the expansion and migration in solid tumors (NCT03198546). In patients with advanced hepatocellular carcinoma, tumors completely disappeared following intratumoral injection of anti-GPC3–7 × 19 CAR-T (174). This study is planned to perform the similar clinical trial on LUSC with the GPC3 expression (NCT03198546). Another Phase IIa trial assessed the efficacy of BMS-813160 (CCR2/5 inhibitor) administered with nivolumab, a PD-1 inhibitor (NCT04123379). In 14 patients with NSCLC, BMS-813160 was co-administered with nivolumab prior to resection, but did not significantly improve the efficacy of the nivolumab treatment (175). In the other Phase I clinical trial (NCT02946671), 12 NSCLC patients administered mogamulizumab, a humanized anti-human CCR4 monoclonal antibody (176). This study exhibited selective depletion of activated Tregs in peripheral blood mononuclear cells and indicated potential immune response induction by mogamulizumab, suggesting this antibody could be used in combination with other immunotherapies to enhance patients’ outcomes. Additionally, two clinical trials employing DC vaccines have been conducted. One Phase I study utilized a CCL21-overexpressing DC vaccine intratumorally in lung cancer patients (NCT01574222) (177). This therapy elicited systemic tumor-antigen-specific immune attack, enhanced tumor infiltration by cytotoxic T cells, and increased PD-L1 expression by tumor cells. Whether PD-L1 upregulation would enhance the effectiveness of PD-1/PD-L1 ICI therapy remains to be evaluated. Another Phase I/randomized Phase II study (NCT01433172) in NSCLC patients compared the GM.CDL vaccine, which recruits and activates DC alone and in combination with CCL21 (178). Although the addition of CCL21 did not yield significant therapeutic benefits overall, it showed promise in one patient with remarkable tumor lymphocyte infiltration, prompting further investigation of CCL21 in combination therapies. Meanwhile, there are studies utilizing chemokines as biomarkers. Early after initiation of anti-PD-1 therapy, an increase in the frequency of the circulating CX3CR1^+^ CD8^+^ T cells is associated with improved response and survival in NSCLC patients (179). Closely related to this study is an ongoing observational trial (NCT06054152) using CX3CR1^+^ CD8^+^ T as a predictor of immunotherapy efficacy for NSCLC patients. CXCR4 is valuable as a diagnostic biomarker, enabling the potential for CXCR4-directed molecular imaging and therapy (180) and the related Phase I study (NCT05557708) has been planned. An exploratory analysis of a phase II study (NCT01439568) (181) assessed the utility of CXCR4 expression in circulating tumor cells as a prognostic biomarker in 89 lung cancer patients (61, 62, 136, 139). This study indicated that positive CXCR4 expression in lung cancer tissue did not significantly impact survival prognosis, highlighting the complexities of utilizing chemokines as standalone therapies in clinical settings despite promising preclinical results in cancer research.

**Table 2:** Clinical trials involving chemokines in lung cancer.

Targeted Chomokine	Molecule	Cancer Type	Phase	Status	Identifier
**CCR2/5**	BMX-813160 (CCR2/5-inhibitor), BMS-986253 (anti-IL-8), nivolumab	NSCLC, hepatocellular carcinoma	II	Completed	NCT04123379
**CCR4**	mogamulizumab (anti-human CCR4 monoclonal antibody) + nivolumab	lung, gastric, esophageal, renal, and oral cancer	I	Completed	NCT02946671
**CCR8**	BAY 3375968 (anti-CCR8 antibody) + pembrolizumab	lung, breast, head and neck cancer, and melanoma	I	Recruiting	NCT05537740
**CXCR1/2**	SX-682 (CXCR1/2 inhibitor) + pembrolizumab	NSCLC	II	Recruiting	NCT05570825
**CXCR4**	LY2510924 (peptide CXCR4 antagonist)	SCLC	II	Completed	NCT01439568
	212-Lead Pentixather (CXCR4-targeted ligand)	lung carcinoid tumor, SCLC, neuroendocrine tumor of the lung	I	Not yet recruiting	NCT05557708
**CXCR5**	CXCR5 modified EGFR CAR-T cells	NSCLC	I	Recruiting	NCT05060796
**CX3CR1**	CX3CR1^+^ CD8^+^ T cells	NSCLC	Principal Test	Active	NCT06054152
**CCL19**	GPC3/TGFβ-CART cells secreting IL7/CCL19 and/or SCFVs	squamous cell lung cancer, hepatocellular carcinoma	I	Recruiting	NCT03198546
**CCL21**	Autologous dendritic cell adenovirus CCL21 vaccine	NSCLC	I	Completed	NCT00601094
				Terminated	NCT01574222
				Active	NCT03546361
	GM.CD40L Vaccine With CCL21	lung cancer, adenocarcinoma	I, II	Completed	NCT01433172
**CXCL12**	CLG (CXCL12 loaded aqueous gel)	solid tumors (lung, endometrium, kidney, glioblastoma, colorectal, and ovary)	Principal Test	Active	NCT05818865

Clinical trials recruiting lung cancer patients for each chemokine receptor and ligand were searched from ClinicalTrials.gov records.

## 5 Conclusion

The advent of cancer immunotherapy has marked a revolutionary shift in cancer treatment, offering a targeted approach to eliminating cancerous tissue while potentially mitigating the adverse effects associated with traditional chemotherapy. This breakthrough has stimulated optimism for both preventing and treating metastatic cancer. However, the TME presents a formidable obstacle, acting as a protective barrier that hampers the efficacy of immunotherapy. Current research endeavors are dedicated to overcoming the challenges posed by immunosuppression and physical barriers within the TME. Chemokines have emerged as pivotal players in augmenting the effectiveness of cancer immunotherapy, orchestrating immune cell signaling within the TME, and facilitating their migration toward cancer cells. Moreover, these chemokines hold promise as valuable biomarkers for prognostication and treatment guidance. Despite abundant preclinical studies, there is a notable scarcity of clinical investigations, likely attributed to the complexity of chemokines and the inherent difficulties in developing them as viable therapeutic agents.

Nevertheless, numerous studies conducted on lung cancer patients underscore the potential of targeting chemokine axes as a promising therapeutic strategy (**Tables 1**, **2**). CXCR2 and CCR2 are the most extensively studied chemokine receptors in preclinical studies of lung cancer. Given that lung tissue is a common site of primary lung cancer development as well as metastasis from tumors outside the lungs, treatments using chemokines such as CXCR4 appear promising. The use of chemokine-related therapies in combination to increase the efficacy of conventional immunotherapy is also being explored in both preclinical and clinical studies, offering promising prospects for enhancing the efficacy of cancer immunotherapy in the future.

## Author contributions

SP: Conceptualization, Funding acquisition, Supervision, Validation, Writing – review & editing. HJ: Conceptualization, Validation, Visualization, Writing – original draft.

## References

[1] Vilgelm AE Richmond A. Chemokines modulate immune surveillance in tumorigenesis, metastasis, and response to immunotherapy. Front Immunol. (2019) 10:333. doi: 10.3389/fimmu.2019.00333 30873179 PMC6400988

[2] Cyster JG. Chemokines and cell migration in secondary lymphoid organs. Science. (1999) 286:2098–102. doi: 10.1126/science.286.5447.2098 10617422

[3] Lahiri A Maji A Potdar PD Singh N Parikh P Bisht B. Lung cancer immunotherapy: progress, pitfalls, and promises. Mol Cancer. (2023) 22:40. doi: 10.1186/s12943-023-01740-y 36810079 PMC9942077

[4] Ligon JA Wessel KM Shah NN Glod J. Adoptive cell therapy in pediatric and young adult solid tumors: current status and future directions. Front Immunol. (2022) 13:846346. doi: 10.3389/fimmu.2022.846346 35273619 PMC8901720

[5] Fucà G Reppel L Landoni E Savoldo B Dotti G. Enhancing chimeric antigen receptor T-cell efficacy in solid tumors. Clin Cancer Res. (2020) 26:2444–51. doi: 10.1158/1078-0432.CCR-19-1835 PMC7269829 32015021

[6] Leiter A Veluswamy RR Wisnivesky JP. The global burden of lung cancer: current status and future trends. Nat Rev Clin Oncol. (2023) 20:624–39. doi: 10.1038/s41571-023-00798-3 37479810

[7] Massarelli E Papadimitrakopoulou V Welsh J Tang C Tsao AS. Immunotherapy in lung cancer. Transl Lung Cancer Res. (2014) 3:53–63. doi: 10.3978/j.issn.2218-6751.2014.01.01 25806281 PMC4367607

[8] Spill F Reynolds DS Kamm RD Zaman MH. Impact of the physical microenvironment on tumor progression and metastasis. Curr Opin Biotechnol. (2016) 40:41–8. doi: 10.1016/j.copbio.2016.02.007 PMC4975620 26938687

[9] Wang Q Shao X Zhang Y Zhu M Wang FXC Mu J. Role of tumor microenvironment in cancer progression and therapeutic strategy. Cancer Med. (2023) 12:11149–65. doi: 10.1002/cam4.5698 PMC10242329 36807772

[10] Junttila MR de Sauvage FJ. Influence of tumour micro-environment heterogeneity on therapeutic response. Nature. (2013) 501:346–54. doi: 10.1038/nature12626 24048067

[11] Ghoshdastider U Rohatgi N Mojtabavi Naeini M Baruah P Revkov E Guo YA. Pan-cancer analysis of ligand–receptor cross-talk in the tumor microenvironment. Cancer Res. (2021) 81:1802–12. doi: 10.1158/0008-5472.CAN-20-2352 33547160

[12] Lei X Lei Y Li J-K Du W-X Li R-G Yang J. Immune cells within the tumor microenvironment: Biological functions and roles in cancer immunotherapy. Cancer Lett. (2020) 470:126–33. doi: 10.1016/j.canlet.2019.11.009 31730903

[13] Koizumi K Hojo S Akashi T Yasumoto K Saiki I. Chemokine receptors in cancer metastasis and cancer cell-derived chemokines in host immune response. Cancer Sci. (2007) 98:1652–8. doi: 10.1111/j.1349-7006.2007.00606.x PMC11159633 17894551

[14] Bule P Aguiar SI Aires-Da-Silva F Dias JNR. Chemokine-directed tumor microenvironment modulation in cancer immunotherapy. Int J Mol Sci. (2021) 22:9804. doi: 10.3390/ijms22189804 34575965 PMC8464715

[15] Marcuzzi E Angioni R Molon B Calì B. Chemokines and chemokine receptors: orchestrating tumor metastasization. Int J Mol Sci. (2018) 20:11. doi: 10.3390/ijms20010096 30591657 PMC6337330

[16] Ljunggren HG Kärre K. In search of the 'missing self': MHC molecules and NK cell recognition. Immunol Today. (1990) 11:237–44. doi: 10.1016/0167-5699(90)90097-S 2201309

[17] Kim J Kim JS Lee HK Kim HS Park EJ Choi JE. CXCR3-deficient natural killer cells fail to migrate to B16F10 melanoma cells. Int Immunopharmacol. (2018) 63:66–73. doi: 10.1016/j.intimp.2018.07.026 30075430

[18] de Andrade LF Lu Y Luoma A Ito Y Pan D Pyrdol JW. Discovery of specialized NK cell populations infiltrating human melanoma metastases. JCI Insight. (2019) 4(23). doi: 10.1172/jci.insight.133103 PMC6962021 31801909

[19] Böttcher JP Bonavita E Chakravarty P Blees H Cabeza-Cabrerizo M Sammicheli S. NK Cells Stimulate Recruitment of cDC1 into the Tumor Microenvironment Promoting Cancer Immune Control. Cell. (2018) 172:1022–1037.e1014. doi: 10.1016/j.cell.2018.01.004 29429633 PMC5847168

[20] Kirchhammer N Trefny MP Natoli M Brücher D Smith SN Werner F. NK cells with tissue-resident traits shape response to immunotherapy by inducing adaptive antitumor immunity. Sci Trans Med. (2022) 14:eabm9043. doi: 10.1126/scitranslmed.abm9043 35857639

[21] Wong JL Berk E Edwards RP Kalinski P. IL-18-primed helper NK cells collaborate with dendritic cells to promote recruitment of effector CD8+ T cells to the tumor microenvironment. Cancer Res. (2013) 73:4653–62. doi: 10.1158/0008-5472.CAN-12-4366 PMC3780558 23761327

[22] Banchereau J Steinman RM. Dendritic cells and the control of immunity. Nature. (1998) 392:245–52. doi: 10.1038/32588 9521319

[23] Vermi W Soncini M Melocchi L Sozzani S Facchetti F. Plasmacytoid dendritic cells and cancer. J Leukoc Biol. (2011) 90:681–90. doi: 10.1189/jlb.0411190 21730085

[24] Bergamaschi C Pandit H Nagy BA Stellas D Jensen SM Bear J. Heterodimeric IL-15 delays tumor growth and promotes intratumoral CTL and dendritic cell accumulation by a cytokine network involving XCL1, IFN-γ, CXCL9 and CXCL10. J Immunother Cancer. (2020) 8. doi: 10.1136/jitc-2020-000599 PMC7254133 32461349

[25] Wang S Wu Q Chen T Su R Pan C Qian J. Blocking CD47 promotes antitumour immunity through CD103+ dendritic cell–NK cell axis in murine hepatocellular carcinoma model. J Hepatol. (2022) 77:467–78. doi: 10.1016/j.jhep.2022.03.011 35367532

[26] Dangaj D Bruand M Grimm AJ Ronet C Barras D Duttagupta PA. Cooperation between constitutive and inducible chemokines enables T cell engraftment and immune attack in solid tumors. Cancer Cell. (2019) 35:885–900.e810. doi: 10.1016/j.ccell.2019.05.004 31185212 PMC6961655

[27] de Mingo Pulido Á Gardner A Hiebler S Soliman H Rugo HS Krummel MF. TIM-3 regulates CD103(+) dendritic cell function and response to chemotherapy in breast cancer. Cancer Cell. (2018) 33:60–74.e66. doi: 10.1016/j.ccell.2017.11.019 29316433 PMC5764109

[28] Xiu W Luo J. CXCL9 secreted by tumor-associated dendritic cells up-regulates PD-L1 expression in bladder cancer cells by activating the CXCR3 signaling. BMC Immunol. (2021) 22:3. doi: 10.1186/s12865-020-00396-3 33407095 PMC7789583

[29] Wu S-Y Zhang S-W Ma D Xiao Y Liu Y Chen L. CCL19+ dendritic cells potentiate clinical benefit of anti-PD-(L)1 immunotherapy in triple-negative breast cancer. Med. (2023) 4:373–393.e378. doi: 10.1016/j.medj.2023.04.008 37201522

[30] Burgoyne P Hayes AJ Cooper RS Le Brocq ML Hansell CAH Campbell JDM. CCR7+ dendritic cells sorted by binding of CCL19 show enhanced Ag-presenting capacity and antitumor potency. J Leukocyte Biol. (2021) 111:1243–51. doi: 10.1002/JLB.5AB0720-446RR 34780080

[31] Dastmalchi F Karachi A Yang C Azari H Sayour EJ Dechkovskaia A. Sarcosine promotes trafficking of dendritic cells and improves efficacy of anti-tumor dendritic cell vaccines via CXC chemokine family signaling. J ImmunoTher Cancer. (2019) 7:321. doi: 10.1186/s40425-019-0809-4 31753028 PMC6873439

[32] Li E Yang X Du Y Wang G Chan DW Wu D. CXCL8 associated dendritic cell activation marker expression and recruitment as indicators of favorable outcomes in colorectal cancer. Front Immunol. (2021) 12:667177. doi: 10.3389/fimmu.2021.667177 34025668 PMC8138166

[33] Slaney CY Kershaw MH Darcy PK. Trafficking of T cells into tumors. Cancer Res. (2014) 74:7168–74. doi: 10.1158/0008-5472.CAN-14-2458 25477332

[34] Zhang J Tao J Gao R-N Wei Z-Y He Y-S Ren C-Y. Cytotoxic T-cell trafficking chemokine profiles correlate with defined mucosal microbial communities in colorectal cancer. Front Immunol. (2021) 12:715559. doi: 10.3389/fimmu.2021.715559 34539647 PMC8442671

[35] Zumwalt TJ Arnold M Goel A Boland CR. Active secretion of CXCL10 and CCL5 from colorectal cancer microenvironments associates with GranzymeB+ CD8+ T-cell infiltration. Oncotarget. (2015) 6:2981–91. doi: 10.18632/oncotarget.v6i5 PMC4413778 25671296

[36] Cózar JM Canton J Tallada M Concha A Cabrera T Garrido F. Analysis of NK cells and chemokine receptors in tumor infiltrating CD4 T lymphocytes in human renal carcinomas. Cancer Immunol Immunother. (2005) 54:858–66. doi: 10.1007/s00262-004-0646-1 PMC11032824 15887015

[37] Huffman AP Lin JH Kim SI Byrne KT Vonderheide RH. CCL5 mediates CD40-driven CD4+ T cell tumor infiltration and immunity. JCI Insight. (2020) 5(10). doi: 10.1172/jci.insight.137263 PMC7259512 32324594

[38] Romero JM Grünwald B Jang G-H Bavi PP Jhaveri A Masoomian M. A four-chemokine signature is associated with a T-cell–inflamed phenotype in primary and metastatic pancreatic cancer. Clin Cancer Res. (2020) 26:1997–2010. doi: 10.1158/1078-0432.CCR-19-2803 31964786

[39] Adler EP Lemken CA Katchen NS Kurt RA. A dual role for tumor-derived chemokine RANTES (CCL5). Immunol Lett. (2003) 90:187–94. doi: 10.1016/j.imlet.2003.09.013 14687724

[40] Chang L-Y Lin Y-C Mahalingam J Huang C-T Chen T-W Kang C-W. Tumor-derived chemokine CCL5 enhances TGF-β–mediated killing of CD8+ T cells in colon cancer by T-regulatory cells. Cancer Res. (2012) 72:1092–102. doi: 10.1158/0008-5472.CAN-11-2493 22282655

[41] Sugasawa H Ichikura T Kinoshita M Ono S Majima T Tsujimoto H. Gastric cancer cells exploit CD4+ cell-derived CCL5 for their growth and prevention of CD8+ cell-involved tumor elimination. Int J Cancer. (2008) 122:2535–41. doi: 10.1002/ijc.23401 18246596

[42] Mellado M de Ana AM Moreno MC Martínez C Rodríguez-Frade JM. A potential immune escape mechanism by melanoma cells through the activation of chemokine-induced T cell death. Curr Biol. (2001) 11:691–6. doi: 10.1016/S0960-9822(01)00199-3 11369232

[43] Chen Y Song Y Du W Gong L Chang H Zou Z. Tumor-associated macrophages: an accomplice in solid tumor progression. J Biomed Sci. (2019) 26:78. doi: 10.1186/s12929-019-0568-z 31629410 PMC6800990

[44] Liu C Yao Z Wang J Zhang W Yang Y Zhang Y. Macrophage-derived CCL5 facilitates immune escape of colorectal cancer cells via the p65/STAT3-CSN5-PD-L1 pathway. Cell Death Differentiation. (2020) 27:1765–81. doi: 10.1038/s41418-019-0460-0 PMC7244707 31802034

[45] Xu W Wu Y Liu W Anwaier A Tian X Su J. Tumor-associated macrophage-derived chemokine CCL5 facilitates the progression and immunosuppressive tumor microenvironment of clear cell renal cell carcinoma. Int J Biol Sci. (2022) 18:4884–900. doi: 10.7150/ijbs.74647 PMC9379407 35982911

[46] Dan H Liu S Liu J Liu D Yin F Wei Z. RACK1 promotes cancer progression by increasing the M2/M1 macrophage ratio via the NF-κB pathway in oral squamous cell carcinoma. Mol Oncol. (2020) 14:795–807. doi: 10.1002/1878-0261.12644 31997535 PMC7138402

[47] Walens A DiMarco AV Lupo R Kroger BR Damrauer JS Alvarez JV. CCL5 promotes breast cancer recurrence through macrophage recruitment in residual tumors. eLife. (2019) 8:e43653. doi: 10.7554/eLife.43653 30990165 PMC6478432

[48] Xie T Fu D-j Li Z-m Lv D-j Song X-L Yu Y-z. CircSMARCC1 facilitates tumor progression by disrupting the crosstalk between prostate cancer cells and tumor-associated macrophages via miR-1322/CCL20/CCR6 signaling. Mol Cancer. (2022) 21:173. doi: 10.1186/s12943-022-01630-9 36045408 PMC9434883

[49] Kadomoto S Izumi K Hiratsuka K Nakano T Naito R Makino T. Tumor-associated macrophages induce migration of renal cell carcinoma cells via activation of the CCL20-CCR6 axis. Cancers. (2020) 12:89. doi: 10.3390/cancers12010089 PMC7073159 32033135

[50] Liu B Jia Y Ma J Wu S Jiang H Cao Y. Tumor-associated macrophage-derived CCL20 enhances the growth and metastasis of pancreatic cancer. Acta Biochim Biophys Sin. (2016) 48:1067–74. doi: 10.1093/abbs/gmw101 27797715

[51] House IG Savas P Lai J Chen AXY Oliver AJ Teo ZL. Macrophage-derived CXCL9 and CXCL10 are required for antitumor immune responses following immune checkpoint blockade. Clin Cancer Res. (2020) 26:487–504. doi: 10.1158/1078-0432.CCR-19-1868 31636098

[52] Chen F Yuan J Yan H Liu H Yin S. Chemokine receptor CXCR3 correlates with decreased M2 macrophage infiltration and favorable prognosis in gastric cancer. BioMed Res Int. (2019) 2019:6832867. doi: 10.1155/2019/6832867 31240220 PMC6556258

[53] Kodama T Koma Y-I Arai N Kido A Urakawa N Nishio M. CCL3–CCR5 axis contributes to progression of esophageal squamous cell carcinoma by promoting cell migration and invasion via Akt and ERK pathways. Lab Invest. (2020) 100:1140–57. doi: 10.1038/s41374-020-0441-4 PMC7438203 32457351

[54] Sheng D Ma W Zhang R Zhou L Deng Q Tu J. Ccl3 enhances docetaxel chemosensitivity in breast cancer by triggering proinflammatory macrophage polarization. J ImmunoTher Cancer. (2022) 10:e003793. doi: 10.1136/jitc-2021-003793 35613826 PMC9134178

[55] Wertel I Surówka J Polak G Barczyński B Bednarek W Jakubowicz-Gil J. Macrophage-derived chemokine CCL22 and regulatory T cells in ovarian cancer patients. Tumor Biol. (2015) 36:4811–7. doi: 10.1007/s13277-015-3133-8 PMC4529457 25647263

[56] Nakanishi T Imaizumi K Hasegawa Y Kawabe T Hashimoto N Okamoto M. Expression of macrophage-derived chemokine (MDC)/CCL22 in human lung cancer. Cancer Immunol Immunother. (2006) 55:1320–9. doi: 10.1007/s00262-006-0133-y PMC11030788 16453150

[57] Chambers AF Groom AC MacDonald IC. Dissemination and growth of cancer cells in metastatic sites. Nat Rev Cancer. (2002) 2:563–72. doi: 10.1038/nrc865 12154349

[58] Bhat AA Nisar S Maacha S Carneiro-Lobo TC Akhtar S Siveen KS. Cytokine-chemokine network driven metastasis in esophageal cancer; promising avenue for targeted therapy. Mol Cancer. (2021) 20:2. doi: 10.1186/s12943-020-01294-3 33390169 PMC7780621

[59] Do HTT Lee CH Cho J. Chemokines and their receptors: multifaceted roles in cancer progression and potential value as cancer prognostic markers. Cancers (Basel). (2020) 12:2. doi: 10.3390/cancers12020287 PMC7072521 31991604

[60] Balkwill F. The significance of cancer cell expression of the chemokine receptor CXCR4. Semin Cancer Biol. (2004) 14:171–9. doi: 10.1016/j.semcancer.2003.10.003 15246052

[61] Müller A Homey B Soto H Ge N Catron D Buchanan ME. Involvement of chemokine receptors in breast cancer metastasis. Nature. (2001) 410:50–6. doi: 10.1038/35065016 11242036

[62] Douglass S Meeson AP Overbeck-Zubrzycka D Brain JG Bennett MR Lamb CA. Breast cancer metastasis: demonstration that FOXP3 regulates CXCR4 expression and the response to CXCL12. J Pathol. (2014) 234:74–85. doi: 10.1002/path.4381 24870556

[63] Yang J Zhang L Jiang Z Ge C Zhao F Jiang J. TCF12 promotes the tumorigenesis and metastasis of hepatocellular carcinoma via upregulation of CXCR4 expression. Theranostics. (2019) 9:5810–27. doi: 10.7150/thno.34973 PMC6735379 31534521

[64] Zhang B Li Y Wu Q Xie L Barwick B Fu C. Acetylation of KLF5 maintains EMT and tumorigenicity to cause chemoresistant bone metastasis in prostate cancer. Nat Commun. (2021) 12:1714. doi: 10.1038/s41467-021-21976-w 33731701 PMC7969754

[65] Khan P Siddiqui JA Kshirsagar PG Venkata RC Maurya SK Mirzapoiazova T. MicroRNA-1 attenuates the growth and metastasis of small cell lung cancer through CXCR4/FOXM1/RRM2 axis. Mol Cancer. (2023) 22:1. doi: 10.1186/s12943-022-01695-6 36597126 PMC9811802

[66] Mortezaee K. CXCL12/CXCR4 axis in the microenvironment of solid tumors: A critical mediator of metastasis. Life Sci. (2020) 249:117534. doi: 10.1016/j.lfs.2020.117534 32156548

[67] Wang M Yang X Wei M Wang Z. The role of CXCL12 axis in lung metastasis of colorectal cancer. J Cancer. (2018) 9:3898–903. doi: 10.7150/jca.26383 PMC6218771 30410593

[68] Urosevic J Blasco MT Llorente A Bellmunt A Berenguer-Llergo A Guiu M. ERK1/2 signaling induces upregulation of ANGPT2 and CXCR4 to mediate liver metastasis in colon cancer. Cancer Res. (2020) 80:4668–80. doi: 10.1158/0008-5472.CAN-19-4028 32816905

[69] Bertolini G Cancila V Milione M Lo Russo G Fortunato O Zaffaroni N. A novel CXCR4 antagonist counteracts paradoxical generation of cisplatin-induced pro-metastatic niches in lung cancer. Mol Ther. (2021) 29:2963–78. doi: 10.1016/j.ymthe.2021.05.014 PMC8530918 34023505

[70] Chaudary N Pintilie M Jelveh S Lindsay P Hill RP Milosevic M. Plerixafor improves primary tumor response and reduces metastases in cervical cancer treated with radio-chemotherapy. Clin Cancer Res. (2017) 23:1242–9. doi: 10.1158/1078-0432.CCR-16-1730 27697997

[71] Gadalla R Hassan H Ibrahim SA Abdullah MS Gaballah A Greve B. Tumor microenvironmental plasmacytoid dendritic cells contribute to breast cancer lymph node metastasis via CXCR4/SDF-1 axis. Breast Cancer Res Treat. (2019) 174:679–91. doi: 10.1007/s10549-019-05129-8 30632021

[72] Toullec A Gerald D Despouy G Bourachot B Cardon M Lefort S. Oxidative stress promotes myofibroblast differentiation and tumour spreading. EMBO Mol Med. (2010) 2:211–30. doi: 10.1002/emmm.201000073 PMC3377319 20535745

[73] Zubrilov I Sagi-Assif O Izraely S Meshel T Ben-Menahem S Ginat R. Vemurafenib resistance selects for highly Malignant brain and lung-metastasizing melanoma cells. Cancer Lett. (2015) 361:86–96. doi: 10.1016/j.canlet.2015.02.041 25725450

[74] Izraely S Klein A Sagi-Assif O Meshel T Tsarfaty G Hoon DS. Chemokine-chemokine receptor axes in melanoma brain metastasis. Immunol Lett. (2010) 130:107–14. doi: 10.1016/j.imlet.2009.12.003 20005902

[75] Klein A Sagi-Assif O Meshel T Telerman A Izraely S Ben-Menachem S. CCR4 is a determinant of melanoma brain metastasis. Oncotarget. (2017) 8:31079–91. doi: 10.18632/oncotarget.v8i19 PMC5458190 28415693

[76] Ou B Zhao J Guan S Feng H Wangpu X Zhu C. Correction: CCR4 promotes metastasis via ERK/NF-κB/MMP13 pathway and acts downstream of TNF-α in colorectal cancer. Oncotarget. (2017) 8:41779. doi: 10.18632/oncotarget.v8i25 28645139 PMC5522284

[77] Zhao H Bo Q Wang W Wang R Li Y Chen S. CCL17-CCR4 axis promotes metastasis via ERK/MMP13 pathway in bladder cancer. J Cell Biochem. (2019) 120:1979–89. doi: 10.1002/jcb.27494 30230587

[78] Cheng X Wu H Jin ZJ Ma D Yuen S Jing XQ. Up-regulation of chemokine receptor CCR4 is associated with Human Hepatocellular Carcinoma Malignant behavior. Sci Rep. (2017) 7:12362. doi: 10.1038/s41598-017-10267-4 28959024 PMC5620046

[79] Nakamura ES Koizumi K Kobayashi M Saitoh Y Arita Y Nakayama T. RANKL-induced CCL22/macrophage-derived chemokine produced from osteoclasts potentially promotes the bone metastasis of lung cancer expressing its receptor CCR4. Clin Exp Metastasis. (2006) 23:9–18. doi: 10.1007/s10585-006-9006-1 16821125

[80] Singh SK Mishra MK Eltoum I-EA Bae S Lillard JW Singh R. CCR5/CCL5 axis interaction promotes migratory and invasiveness of pancreatic cancer cells. Sci Rep. (2018) 8:1323. doi: 10.1038/s41598-018-19643-0 29358632 PMC5778036

[81] Aldinucci D Lorenzon D Cattaruzza L Pinto A Gloghini A Carbone A. Expression of CCR5 receptors on Reed–Sternberg cells and Hodgkin lymphoma cell lines: Involvement of CCL5/Rantes in tumor cell growth and microenvironmental interactions. Int J Cancer. (2008) 122:769–76. doi: 10.1002/ijc.23119 17935139

[82] Casagrande N Borghese C Visser L Mongiat M Colombatti A Aldinucci D. CCR5 antagonism by maraviroc inhibits Hodgkin lymphoma microenvironment interactions and xenograft growth. Haematologica. (2019) 104:564–75. doi: 10.3324/haematol.2018.196725 PMC6395337 30309853

[83] Singh SK Mishra MK Rivers BM Gordetsky JB Bae S Singh R. Biological and clinical significance of the CCR5/CCL5 axis in hepatocellular carcinoma. Cancers. (2020) 12:883. doi: 10.3390/cancers12040883 32260550 PMC7226629

[84] Pervaiz A Zepp M Georges R Bergmann F Mahmood S Faiza S. Antineoplastic effects of targeting CCR5 and its therapeutic potential for colorectal cancer liver metastasis. J Cancer Res Clin Oncol. (2021) 147:73–91. doi: 10.1007/s00432-020-03382-9 32902795 PMC7810651

[85] Liu J Wang C Ma X Tian Y Wang C Fu Y. High expression of CCR5 in melanoma enhances epithelial–mesenchymal transition and metastasis via TGFβ1. J Pathol. (2019) 247:481–93. doi: 10.1002/path.5207 30474221

[86] González-Arriagada WA Coletta RD Lozano-Burgos C García C Maripillán J Alcayaga-Miranda F. CR5/CCL5 axis is linked to a poor outcome, and inhibition reduces metastasis in oral squamous cell carcinoma. J Cancer Res Clin Oncol. (2023) 149:17335–46. doi: 10.1007/s00432-023-05443-1 PMC11797508 37831273

[87] Tang C-H Yamamoto A Lin Y-T Fong Y-C Tan T-W. Involvement of matrix metalloproteinase-3 in CCL5/CCR5 pathway of chondrosarcomas metastasis. Biochem Pharmacol. (2010) 79:209–17. doi: 10.1016/j.bcp.2009.08.006 19682436

[88] Wu Y Li Y-Y Matsushima K Baba T Mukaida N. CCL3-CCR5 axis regulates intratumoral accumulation of leukocytes and fibroblasts and promotes angiogenesis in murine lung metastasis process1. J Immunol. (2008) 181:6384–93. doi: 10.4049/jimmunol.181.9.6384 18941229

[89] Kodama T Koma YI Arai N Kido A Urakawa N Nishio M. CCL3-CCR5 axis contributes to progression of esophageal squamous cell carcinoma by promoting cell migration and invasion via Akt and ERK pathways. Lab Invest. (2020) 100:1140–57. doi: 10.1038/s41374-020-0441-4 PMC7438203 32457351

[90] Sasaki S Baba T Nishimura T Hayakawa Y Hashimoto S-i Gotoh N. Essential roles of the interaction between cancer cell-derived chemokine, CCL4, and intra-bone CCR5-expressing fibroblasts in breast cancer bone metastasis. Cancer Lett. (2016) 378:23–32. doi: 10.1016/j.canlet.2016.05.005 27177471

[91] Lee D Shin K-J Kim DW Yoon K-A Choi Y-J Lee BNR. CCL4 enhances preosteoclast migration and its receptor CCR5 downregulation by RANKL promotes osteoclastogenesis. Cell Death Dis. (2018) 9:495. doi: 10.1038/s41419-018-0562-5 29717113 PMC5931580

[92] Fan L Reilly CR Luo Y Dorf ME Lo D. Cutting edge: ectopic expression of the chemokine TCA4/SLC is sufficient to trigger lymphoid neogenesis. J Immunol. (2000) 164:3955–9. doi: 10.4049/jimmunol.164.8.3955 10754285

[93] López-Giral S Quintana NE Cabrerizo M Alfonso-Pérez M Sala-Valdés M De Soria VG. Chemokine receptors that mediate B cell homing to secondary lymphoid tissues are highly expressed in B cell chronic lymphocytic leukemia and non-Hodgkin lymphomas with widespread nodular dissemination. J Leukoc Biol. (2004) 76:462–71. doi: 10.1189/jlb.1203652 15155773

[94] Sperveslage J Frank S Heneweer C Egberts J Schniewind B Buchholz M. Lack of CCR7 expression is rate limiting for lymphatic spread of pancreatic ductal adenocarcinoma. Int J Cancer. (2012) 131:E371–81. doi: 10.1002/ijc.26502 22020953

[95] Zhong G Chen L Yin R Qu Y Bao Y Xiao Q. Chemokine (C−C motif) ligand 21/C−C chemokine receptor type 7 triggers migration and invasion of human lung cancer cells by epithelial−mesenchymal transition via the extracellular signal−regulated kinase signaling pathway. Mol Med Rep. (2017) 15:4100–8. doi: 10.3892/mmr.2017.6534 PMC5436267 28487957

[96] Yu J Tao S Hu P Wang R Fang C Xu Y. CCR7 promote lymph node metastasis via regulating VEGF-C/D-R3 pathway in lung adenocarcinoma. J Cancer. (2017) 8:2060–8. doi: 10.7150/jca.19069 PMC5559968 28819407

[97] Zhang S Wang H Xu Z Bai Y Xu L. Lymphatic metastasis of NSCLC involves chemotaxis effects of lymphatic endothelial cells through the CCR7–CCL21 axis modulated by TNF-α. Genes. (2020) 11:1309. doi: 10.3390/genes11111309 33158173 PMC7694274

[98] Mohammed MM Shaker O Ramzy MM Gaber SS Kamel HS Abed El Baky MF. The relation between ACKR4 and CCR7 genes expression and breast cancer metastasis. Life Sci. (2021) 279:119691. doi: 10.1016/j.lfs.2021.119691 34102193

[99] Emmett MS Lanati S Dunn DBA Stone OA Bates DO. CCR7 mediates directed growth of melanomas towards lymphatics. Microcirculation. (2011) 18:172–82. doi: 10.1111/j.1549-8719.2010.00074.x PMC3287291 21166932

[100] Shi M Chen D Yang D Liu X-y. CCL21-CCR7 promotes the lymph node metastasis of esophageal squamous cell carcinoma by up-regulating MUC1. J Exp Clin Cancer Res. (2015) 34:149. doi: 10.1186/s13046-015-0268-9 26667143 PMC4678529

[101] Rachmadi L Laelasari E Susanto YDB Kusmardi K. MMP-9 and CCR7 as possible predictors of lymph node metastasis in laryngeal squamous cell carcinoma. Iran J Pathol. (2023) 18:156–64. doi: 10.30699/ijp.2023.563014.2986 PMC10439748 37600570

[102] Dai Y Tong R Guo H Yu T Wang C. Association of CXCR4, CCR7, VEGF-C and VEGF-D expression with lymph node metastasis in patients with cervical cancer. Eur J Obstetrics Gynecol Reprod Biol. (2017) 214:178–83. doi: 10.1016/j.ejogrb.2017.04.043 28535405

[103] Issa A Le TX Shoushtari AN Shields JD Swartz MA. Vascular endothelial growth factor-C and C-C chemokine receptor 7 in tumor cell–lymphatic cross-talk promote invasive phenotype. Cancer Res. (2008) 69:349–57. doi: 10.1158/0008-5472.Can-08-1875 19118020

[104] Maolake A Izumi K Natsagdorj A Iwamoto H Kadomoto S Makino T. Tumor necrosis factor-α induces prostate cancer cell migration in lymphatic metastasis through CCR7 upregulation. Cancer Sci. (2018) 109:1524–31. doi: 10.1111/cas.13586 PMC5980342 29575464

[105] De Larco JE Wuertz BRK Rosner KA Erickson SA Gamache DE Manivel JC. A potential role for interleukin-8 in the metastatic phenotype of breast carcinoma cells. Am J Pathol. (2001) 158:639–46. doi: 10.1016/S0002-9440(10)64005-9 PMC1850317 11159200

[106] Desai S Laskar S Pandey BN. Autocrine IL-8 and VEGF mediate epithelial–mesenchymal transition and invasiveness via p38/JNK-ATF-2 signalling in A549 lung cancer cells. Cell Signalling. (2013) 25:1780–91. doi: 10.1016/j.cellsig.2013.05.025 23714383

[107] Li Z Liu J Li L Shao S Wu J Bian L. Epithelial mesenchymal transition induced by the CXCL9/CXCR3 axis through AKT activation promotes invasion and metastasis in tongue squamous cell carcinoma. Oncol Rep. (2018) 39:1356–68. doi: 10.3892/or.2017.6169 29286143

[108] Ding Q Xia Y Ding S Lu P Sun L Liu M. An alternatively spliced variant of CXCR3 mediates the metastasis of CD133+ liver cancer cells induced by CXCL9. Oncotarget. (2016) 7:14405–14. doi: 10.18632/oncotarget.v7i12 PMC4924724 26883105

[109] Chao C-C Lee W-F Wang S-W Chen P-C Yamamoto A Chang T-M. CXC chemokine ligand-13 promotes metastasis via CXCR5-dependent signaling pathway in non-small cell lung cancer. J Cell Mol Med. (2021) 25:9128–40. doi: 10.1111/jcmm.16743 PMC8500967 34427969

[110] Yang J Lv X Chen J Xie C Xia W Jiang C. CCL2-CCR2 axis promotes metastasis of nasopharyngeal carcinoma by activating ERK1/2-MMP2/9 pathway. Oncotarget. (2016) 7:15632–47. doi: 10.18632/oncotarget.v7i13 PMC4941266 26701209

[111] Roblek M Protsyuk D Becker PF Stefanescu C Gorzelanny C Glaus Garzon JF. CCL2 is a vascular permeability factor inducing CCR2-dependent endothelial retraction during lung metastasis. Mol Cancer Res. (2019) 17:783–93. doi: 10.1158/1541-7786.MCR-18-0530 PMC6445360 30552233

[112] Kapur N Mir H Clark Iii CE Krishnamurti U Beech DJ Lillard JW. CCR6 expression in colon cancer is associated with advanced disease and supports epithelial-to-mesenchymal transition. Br J Cancer. (2016) 114:1343–51. doi: 10.1038/bjc.2016.113 PMC4984452 27149649

[113] Zhang XP Hu ZJ Meng AH Duan GC Zhao QT Yang J. Role of CCL20/CCR6 and the ERK signaling pathway in lung adenocarcinoma. Oncol Lett. (2017) 14:8183–9. doi: 10.3892/ol.2017.7253 PMC5727607 29250193

[114] Mazur G Jaskuła E Kryczek I Dłubek D Butrym A Wróbel T. Proinflammatory chemokine gene expression influences survival of patients with non-Hodgkin's lymphoma. Folia Histochem Cytobiol. (2011) 49:240–7. doi: 10.5603/FHC.2011.0033 21744323

[115] Prabhakaran S Rizk VT Ma Z Cheng C-H Berglund AE Coppola D. Evaluation of invasive breast cancer samples using a 12-chemokine gene expression score: correlation with clinical outcomes. Breast Cancer Res. (2017) 19:71. doi: 10.1186/s13058-017-0864-z 28629479 PMC5477261

[116] Fan T Liu Y Liu H Wang L Tian H Zheng Y. Comprehensive analysis of a chemokine- and chemokine receptor family-based signature for patients with lung adenocarcinoma. Cancer Immunol Immunother. (2021) 70:3651–67. doi: 10.1007/s00262-021-02944-1 PMC10992915 33977344

[117] Solari R Pease JE Begg M. Chemokine receptors as therapeutic targets: Why aren’t there more drugs? Eur J Pharmacol. (2015) 746:363–7. doi: 10.1016/j.ejphar.2014.06.060 25016087

[118] Schall TJ Proudfoot AEI. Overcoming hurdles in developing successful drugs targeting chemokine receptors. Nat Rev Immunol. (2011) 11:355–63. doi: 10.1038/nri2972 21494268

[119] Di Marino D Conflitti P Motta S Limongelli V. Structural basis of dimerization of chemokine receptors CCR5 and CXCR4. Nat Commun. (2023) 14:6439. doi: 10.1038/s41467-023-42082-z 37833254 PMC10575954

[120] Nichols WG Steel HM Bonny T Adkison K Curtis L Millard J. Hepatotoxicity observed in clinical trials of aplaviroc (GW873140). Antimicrobial Agents Chemother. (2008) 52:858–65. doi: 10.1128/AAC.00821-07 PMC2258506 18070967

[121] Sterner RC Sterner RM. CAR-T cell therapy: current limitations and potential strategies. Blood Cancer J. (2021) 11:69. doi: 10.1038/s41408-021-00459-7 33824268 PMC8024391

[122] Li H Harrison EB Li H Hirabayashi K Chen J Li Q-X. Targeting brain lesions of non-small cell lung cancer by enhancing CCL2-mediated CAR-T cell migration. Nat Commun. (2022) 13:2154. doi: 10.1038/s41467-022-29647-0 35443752 PMC9021299

[123] Adachi K Kano Y Nagai T Okuyama N Sakoda Y Tamada K. IL-7 and CCL19 expression in CAR-T cells improves immune cell infiltration and CAR-T cell survival in the tumor. Nat Biotechnol. (2018) 36:346–51. doi: 10.1038/nbt.4086 29505028

[124] Smith DM Schafer JR Tullius B Witkam L Paust S. Natural killer cells for antiviral therapy. Sci Trans Med. (2023) 15:eabl5278. doi: 10.1126/scitranslmed.abl5278 36599006

[125] Schomer NT Jiang ZK Lloyd MI Klingemann H Boissel L. CCR7 expression in CD19 chimeric antigen receptor-engineered natural killer cells improves migration toward CCL19-expressing lymphoma cells and increases tumor control in mice with human lymphoma. Cytotherapy. (2022) 24:827–34. doi: 10.1016/j.jcyt.2022.02.006 35400595

[126] Jamali A Hadjati J Madjd Z Mirzaei HR Thalheimer FB Agarwal S. Highly efficient generation of transgenically augmented CAR NK cells overexpressing CXCR4. Front Immunol. (2020) 11:2028. doi: 10.3389/fimmu.2020.02028 32983147 PMC7483584

[127] Wang G Zhang Z Zhong K Wang Z Yang N Tang X. CXCL11-armed oncolytic adenoviruses enhance CAR-T cell therapeutic efficacy and reprogram tumor microenvironment in glioblastoma. Mol Ther. (2023) 31:134–53. doi: 10.1016/j.ymthe.2022.08.021 PMC9840126 36056553

[128] Li F Sheng Y Hou W Sampath P Byrd D Thorne S. CCL5-armed oncolytic virus augments CCR5-engineered NK cell infiltration and antitumor efficiency. J ImmunoTher Cancer. (2020) 8:e000131. doi: 10.1136/jitc-2019-000131 32098828 PMC7057442

[129] Feigl FF Stahringer A Peindl M Dandekar G Koehl U Fricke S. Efficient redirection of NK cells by genetic modification with chemokine receptors CCR4 and CCR2B. Int J Mol Sci. (2023) 24:3129. doi: 10.3390/ijms24043129 36834542 PMC9967507

[130] Shiravand Y Khodadadi F Kashani SMA Hosseini-Fard SR Hosseini S Sadeghirad H. Immune checkpoint inhibitors in cancer therapy. Curr Oncol. (2022) 29:3044–60. doi: 10.3390/curroncol29050247 PMC9139602 35621637

[131] Martinez-Usatorre A Kadioglu E Boivin G Cianciaruso C Guichard A Torchia B. Overcoming microenvironmental resistance to PD-1 blockade in genetically engineered lung cancer models. Sci Transl Med. (2021) 13(606). doi: 10.1126/scitranslmed.abd1616 PMC7612153 34380768

[132] Sun L Bleiberg B Hwang W-T Marmarelis ME Langer CJ Singh A. Association between duration of immunotherapy and overall survival in advanced non–small cell lung cancer. JAMA Oncol. (2023) 9:1075–82. doi: 10.1001/jamaoncol.2023.1891 PMC10240399 37270700

[133] Herbst RS Garon EB Kim DW Cho BC Perez-Gracia JL Han JY. Long-term outcomes and retreatment among patients with previously treated, programmed death-ligand 1−Positive, advanced non−Small-cell lung cancer in the KEYNOTE-010 study. J Clin Oncol. (2020) 38:1580–90. doi: 10.1200/JCO.19.02446 32078391

[134] Reck M Rodríguez-Abreu D Robinson AG Hui R Csőszi T Fülöp A. Five-year outcomes with pembrolizumab versus chemotherapy for metastatic non-small-cell lung cancer with PD-L1 tumor proportion score ≥ 50. J Clin Oncol. (2021) 39:2339–49. doi: 10.1200/JCO.21.00174 PMC8280089 33872070

[135] Sorin M Karimi E Rezanejad M Yu MW Desharnais L McDowell SAC. Single-cell spatial landscape of immunotherapy response reveals mechanisms of CXCL13 enhanced antitumor immunity. J ImmunoTher Cancer. (2023) 11:e005545. doi: 10.1136/jitc-2022-005545 36725085 PMC9896310

[136] Bockorny B Semenisty V Macarulla T Borazanci E Wolpin BM Stemmer SM. BL-8040, a CXCR4 antagonist, in combination with pembrolizumab and chemotherapy for pancreatic cancer: the COMBAT trial. Nat Med. (2020) 26:878–85. doi: 10.1038/s41591-020-0880-x 32451495

[137] Ackun-Farmmer MA Soto CA Lesch ML Byun D Yang L Calvi LM. Reduction of leukemic burden via bone-targeted nanoparticle delivery of an inhibitor of C-chemokine (C-C motif) ligand 3 (CCL3) signaling. FASEB J. (2021) 35:e21402. doi: 10.1096/fj.202000938RR 33724567 PMC8594422

[138] Alghamri MS Banerjee K Mujeeb AA Mauser A Taher A Thalla R. Systemic delivery of an adjuvant CXCR4-CXCL12 signaling inhibitor encapsulated in synthetic protein nanoparticles for glioma immunotherapy. ACS Nano. (2022) 16:8729–50. doi: 10.1021/acsnano.1c07492 PMC9649873 35616289

[139] Li Z Wang Y Shen Y Qian C Oupicky D Sun M. Targeting pulmonary tumor microenvironment with CXCR4-inhibiting nanocomplex to enhance anti–PD-L1 immunotherapy. Sci Adv. (2020) 6:eaaz9240. doi: 10.1126/sciadv.aaz9240 32440550 PMC7228744

[140] Jiang X Wang C Fitch S Yang F. Targeting tumor hypoxia using nanoparticle-engineered CXCR4-overexpressing adipose-derived stem cells. Theranostics. (2018) 8:1350–60. doi: 10.7150/thno.22736 PMC5835941 29507625

[141] Gao C Cheng K Li Y Gong R Zhao X Nie G. Injectable immunotherapeutic hydrogel containing RNA-loaded lipid nanoparticles reshapes tumor microenvironment for pancreatic cancer therapy. Nano Lett. (2022) 22:8801–9. doi: 10.1021/acs.nanolett.2c01994 36251255

[142] Altorki NK Markowitz GJ Gao D Port JL Saxena A Stiles B. The lung microenvironment: an important regulator of tumour growth and metastasis. Nat Rev Cancer. (2019) 19:9–31. doi: 10.1038/s41568-018-0081-9 30532012 PMC6749995

[143] Cheng CC Chang YF Ho AS Sie ZL Chang JS Peng CL. Irradiation mediates IFNα and CXCL9 expression in non-small cell lung cancer to stimulate CD8(+) T cells activity and migration toward tumors. Biomedicines. (2021) 9:10. doi: 10.3390/biomedicines9101349 PMC8533192 34680466

[144] Yang F Zhang S Meng Q Zhou F Pan B Liu F. CXCR1 correlates to poor outcomes of EGFR-TKI against advanced non-small cell lung cancer by activating chemokine and JAK/STAT pathway. Pulmonary Pharmacol Ther. (2021) 67:102001. doi: 10.1016/j.pupt.2021.102001 33582208

[145] Unver N. Identification of the dominant angiogenic CXCL class chemokines associated with non-small cell lung cancer via bioinformatics tools. Med Oncol. (2021) 38:68. doi: 10.1007/s12032-021-01517-7 33983509

[146] White ES Flaherty KR Carskadon S Brant A Iannettoni MD Yee J. Macrophage migration inhibitory factor and CXC chemokine expression in non-small cell lung cancer: role in angiogenesis and prognosis1. Clin Cancer Res. (2003) 9:853–60. 12576459

[147] Keane MP Belperio JA Xue YY Burdick MD Strieter RM. Depletion of CXCR2 inhibits tumor growth and angiogenesis in a murine model of lung cancer1. J Immunol. (2004) 172:2853–60. doi: 10.4049/jimmunol.172.5.2853 14978086

[148] Cheng Y Mo F Li Q Han X Shi H Chen S. Targeting CXCR2 inhibits the progression of lung cancer and promotes therapeutic effect of cisplatin. Mol Cancer. (2021) 20:62. doi: 10.1186/s12943-021-01355-1 33814009 PMC8019513

[149] Hao B Zhang Z Lu Z Xiong J Fan T Song C. Single-cell RNA sequencing analysis revealed cellular and molecular immune profiles in lung squamous cell carcinoma. Trans Oncol. (2023) 27:101568. doi: 10.1016/j.tranon.2022.101568 PMC9586982 36270103

[150] Lv M Xu Y Tang R Ren J Shen S Chen Y. miR141–CXCL1–CXCR2 signaling–induced treg recruitment regulates metastases and survival of non–small cell lung cancer. Mol Cancer Ther. (2014) 13:3152–62. doi: 10.1158/1535-7163.MCT-14-0448 25349304

[151] Hu S Zha Y Yang W Cui K Cheng M. Dysregulation of NK and CD8^+^T cells by the microbiota promotes the progression of lung cancer. J Immunol Res. (2022) 2022:7057089. doi: 10.1155/2022/7057089 36033391 PMC9410859

[152] Hong SH Kang N Kim O Hong SA Park J Kim J. EGFR-tyrosine kinase inhibitors induced activation of the autocrine CXCL10/CXCR3 pathway through crosstalk between the tumor and the microenvironment in EGFR-mutant lung cancer. Cancers. (2023) 15:124. doi: 10.3390/cancers15010124 PMC9817815 36612121

[153] Guo W Huai Q Zhou B Guo L Sun L Xue X. Comprehensive analysis of the immunological implication and prognostic value of CXCR4 in non-small cell lung cancer. Cancer Immunol Immunother. (2023) 72:1029–45. doi: 10.1007/s00262-022-03298-y PMC10025233 36308553

[154] Qiu L Xu Y Xu H Yu B. The clinicopathological and prognostic value of CXCR4 expression in patients with lung cancer: a meta-analysis. BMC Cancer. (2022) 22:681. doi: 10.1186/s12885-022-09756-1 35729596 PMC9210617

[155] Taromi S Kayser G Catusse J von Elverfeldt D Reichardt W Braun F. CXCR4 antagonists suppress small cell lung cancer progression. Oncotarget. (2016) 7:85185–95. doi: 10.18632/oncotarget.v7i51 PMC5356728 27835905

[156] Schmall A Al-tamari HM Herold S Kampschulte M Weigert A Wietelmann A. Macrophage and cancer cell cross-talk via CCR2 and CX3CR1 is a fundamental mechanism driving lung cancer. Am J Respir Crit Care Med. (2015) 191:437–47. doi: 10.1164/rccm.201406-1137OC 25536148

[157] Zheng Y Wang Z Wei S Liu Z Chen G. Epigenetic silencing of chemokine CCL2 represses macrophage infiltration to potentiate tumor development in small cell lung cancer. Cancer Lett. (2021) 499:148–63. doi: 10.1016/j.canlet.2020.11.034 33253790

[158] Fridlender ZG Kapoor V Buchlis G Cheng G Sun J Wang L-CS. Monocyte chemoattractant protein–1 blockade inhibits lung cancer tumor growth by altering macrophage phenotype and activating CD8+ Cells. Am J Respir Cell Mol Biol. (2011) 44:230–7. doi: 10.1165/rcmb.2010-0080OC PMC3049234 20395632

[159] Wang Y Zhang X Yang L Xue J Hu G. Blockade of CCL2 enhances immunotherapeutic effect of anti-PD1 in lung cancer. J Bone Oncol. (2018) 11:27–32. doi: 10.1016/j.jbo.2018.01.002 29892522 PMC5993943

[160] Chidimatsu H Tsunedomi R Nakagami Y Xu M Nakajima M Nakashima-Nakasuga C. Serum CCL7 is a novel prognostic biomarker of metastatic colorectal cancer. Anticancer Res. (2023) 43:105–14. doi: 10.21873/anticanres.16139 36585204

[161] Lee YS Kim SY Song SJ Hong HK Lee Y Oh BY. Crosstalk between CCL7 and CCR3 promotes metastasis of colon cancer cells via ERK-JNK signaling pathways. Oncotarget. (2016) 7:36842–53. doi: 10.18632/oncotarget.v7i24 PMC5095043 27167205

[162] Kanyomse Q Le X Tang J Dai F Mobet Y Chen C. KLF15 suppresses tumor growth and metastasis in Triple-Negative Breast Cancer by downregulating CCL2 and CCL7. Sci Rep. (2022) 12:19026. doi: 10.1038/s41598-022-23750-4 36347994 PMC9643362

[163] Mise Y Hamanishi J Daikoku T Takamatsu S Miyamoto T Taki M. Immunosuppressive tumor microenvironment in uterine serous carcinoma via CCL7 signal with myeloid-derived suppressor cells. Carcinogenesis. (2022) 43:647–58. doi: 10.1093/carcin/bgac032 35353883

[164] Zhang M Yang W Wang P Deng Y Dong Y-T Liu F-F. CCL7 recruits cDC1 to promote antitumor immunity and facilitate checkpoint immunotherapy to non-small cell lung cancer. Nat Commun. (2020) 11:6119. doi: 10.1038/s41467-020-19973-6 33257678 PMC7704643

[165] Dong H-P Li Y Tang Z Wang P Zhong B Chu Q. Combined targeting of CCL7 and Flt3L to promote the expansion and infiltration of cDC1s in tumors enhances T-cell activation and anti-PD-1 therapy effectiveness in NSCLC. Cell Mol Immunol. (2023) 20:850–3. doi: 10.1038/s41423-023-00991-5 PMC10310796 36894615

[166] Melese ES Franks E Cederberg RA Harbourne BT Shi R Wadsworth BJ. CCL5 production in lung cancer cells leads to an altered immune microenvironment and promotes tumor development. OncoImmunology. (2022) 11:2010905. doi: 10.1080/2162402X.2021.2010905 35481284 PMC9038050

[167] Larroquette M Guegan J-P Besse B Cousin S Brunet M Moulec SL. Spatial transcriptomics of macrophage infiltration in non-small cell lung cancer reveals determinants of sensitivity and resistance to anti-PD1/PD-L1 antibodies. J ImmunoTher Cancer. (2022) 10:e003890. doi: 10.1136/jitc-2021-003890 35618288 PMC9125754

[168] Korbecki J Olbromski M Dzięgiel P. CCL18 in the progression of cancer. Int J Mol Sci. (2020) 21:7955. doi: 10.3390/ijms21217955 33114763 PMC7663205

[169] Shi L Zhang B Sun X Zhang X Lv S Li H. CC chemokine ligand 18(CCL18) promotes migration and invasion of lung cancer cells by binding to Nir1 through Nir1-ELMO1/DOC180 signaling pathway. Mol Carcinog. (2016) 55:2051–62. doi: 10.1002/mc.22450 26756176

[170] Plönes T Krohn A Burger M Veelken H Passlick B Müller-Quernheim J. Serum level of CC-chemokine ligand 18 is increased in patients with non-small-cell lung cancer and correlates with survival time in adenocarcinomas. PloS One. (2012) 7:e41746. doi: 10.1371/journal.pone.0041746 22848587 PMC3404958

[171] Schmid S Le U-T Haager B Mayer O Dietrich I Elze M. Local concentrations of CC-chemokine-ligand 18 correlate with tumor size in non-small cell lung cancer and are elevated in lymph node-positive disease. Anticancer Res. (2016) 36:4667–71. doi: 10.21873/anticanres 27630310

[172] Schmid S Csanadi A Kozhuharov N Tchudjin M Kayser C Rawluk J. CC-chemokine ligand 18 is an independent prognostic marker in lymph node-positive non-small cell lung cancer. Anticancer Res. (2018) 38:3913–8. doi: 10.21873/anticanres.12676 29970512

[173] Wu J Liu X Wu J Lou C Zhang Q Chen H. CXCL12 derived from CD248-expressing cancer-associated fibroblasts mediates M2-polarized macrophages to promote nonsmall cell lung cancer progression. Biochim Biophys Acta Mol Basis Dis. (2022) 1868:166521. doi: 10.1016/j.bbadis.2022.166521 35985448

[174] Pang N Shi J Qin L Chen A Tang Y Yang H. IL-7 and CCL19-secreting CAR-T cell therapy for tumors with positive glypican-3 or mesothelin. J Hematol Oncol. (2021) 14:118. doi: 10.1186/s13045-021-01128-9 34325726 PMC8323212

[175] Venturini NJ Hamon P Ward S Fiel MI Beasley MB Kim E. (2023). Targeting myeloid cells in non-small cell lung cancer and hepatocellular carcinoma: A window-of-opportunity trial of nivolumab with BMS-813160 (CCR2/5i) or BMS-986253 (anti-IL8), in: Abstract Book of the ESMO Immuno-Oncology Congress 2023. Geneva Switzerland: Immuno-oncology and technology, Vol. 20. p. 3. doi: 10.1016/j.iotech.2023.100629

[176] Kurose K Ohue Y Wada H Iida S Ishida T Kojima T. Phase ia study of foxP3+ CD4 treg depletion by infusion of a humanized anti-CCR4 antibody, KW-0761, in cancer patients. Clin Cancer Res. (2015) 21:4327–36. doi: 10.1158/1078-0432.CCR-15-0357 26429981

[177] Lee JM Lee M-H Garon E Goldman JW Salehi-Rad R Baratelli FE. Phase I trial of intratumoral injection of CCL21 gene–modified dendritic cells in lung cancer elicits tumor-specific immune responses and CD8+ T-cell infiltration. Clin Cancer Res. (2017) 23:4556–68. doi: 10.1158/1078-0432.CCR-16-2821 PMC5599263 28468947

[178] Gray JE Chiappori A Williams CC Tanvetyanon T Haura EB Creelan BC. A phase I/randomized phase II study of GM.CD40L vaccine in combination with CCL21 in patients with advanced lung adenocarcinoma. Cancer Immunol Immunother. (2018) 67:1853–62. doi: 10.1007/s00262-018-2236-7 PMC6244998 30209589

[179] Yamauchi T Hoki T Oba T Jain V Chen H Attwood K. T-cell CX3CR1 expression as a dynamic blood-based biomarker of response to immune checkpoint inhibitors. Nat Commun. (2021) 12:1402. doi: 10.1038/s41467-021-21619-0 33658501 PMC7930182

[180] Hartrampf PE Kosmala A Serfling SE Bundschuh L Higuchi T Lapa C. Interobserver agreement rates on C-X-C motif chemokine receptor 4-directed molecular imaging and therapy. Clin Nucl Med. (2023) 48:483–8. doi: 10.1097/RLU.0000000000004629 PMC10184817 36947793

[181] Salgia R Weaver RW McCleod M Stille JR Yan SB Roberson S. Prognostic and predictive value of circulating tumor cells and CXCR4 expression as biomarkers for a CXCR4 peptide antagonist in combination with carboplatin-etoposide in small cell lung cancer: exploratory analysis of a phase II study. Investigational New Drugs. (2017) 35:334–44. doi: 10.1007/s10637-017-0446-z PMC5418321 28299514

